# MRPL13 enhances mitochondrial function and promotes tumor progression in ovarian cancer by inhibiting mPTP opening via SLC25A6

**DOI:** 10.1038/s41419-025-07953-x

**Published:** 2025-08-21

**Authors:** Ouxuan Liu, Yuexin Hu, Shuang Wang, Xin Nie, Yuxuan Wang, Xiangcheng Fan, Kai Zeng, Xiao Li, Bingying Liu, Bei Lin

**Affiliations:** 1 Department of Obstetrics and Gynecology, Shengjing Hospital of China Medical University, Shenyang, Liaoning China; 2 Key Laboratory of Obstetrics and Gynecology of Higher Education of Liaoning Province, Key Laboratory of Gynecologic Oncology of Liaoning Province, Shenyang, Liaoning China; 3 Department of Obstetrics and Gynecology, Tianjin Central Hospital of Gynecology Obstetrics, Tianjin, China

**Keywords:** Ovarian cancer, Oncogenes, Cell death, Cancer metabolism

## Abstract

Tumor cells typically exhibit dysregulation of mitochondrial energy metabolism and cell death. The role of mitochondrial function in ovarian cancer (OC) progression has garnered substantial attention, yet its precise molecular mechanisms remain elusive. Mitochondrial ribosomal protein L13 (MRPL13), involved in the translation of oxidative phosphorylation (OXPHOS) complex subunits, plays a critical role in regulating mitochondrial function. Our study demonstrated that MRPL13 is highly expressed in OC tissues and correlated with poor prognosis. Both in vitro and in vivo experiments confirmed that MRPL13 overexpression significantly promotes the malignant biological behavior of OC, while MRPL13 knockdown induces the opposite phenotype. Moreover, MRPL13 knockdown impairs mitochondrial function in OC cells, leading to decreased OXPHOS and ATP levels, increased reactive oxygen species (ROS) generation, mitochondrial depolarization, aberrant opening of the mitochondrial permeability transition pore (mPTP), and mitochondrial structural damage. Mechanistically, MRPL13 specifically interacts with SLC25A6 and facilitates its degradation via lysine (K)48-linked ubiquitination. MRPL13 inhibits mPTP opening by accelerating the degradation of SLC25A6, thereby preventing cytochrome c release into the cytoplasm, inhibiting cell death, and enhancing mitochondrial function. In conclusion, our study elucidates the mechanism by which the MRPL13-SLC25A6 axis enhances mitochondrial function and promotes tumor progression in OC by inhibiting mPTP opening, suggesting that MRPL13 holds significant potential for prognostic evaluation and targeted therapy in OC.

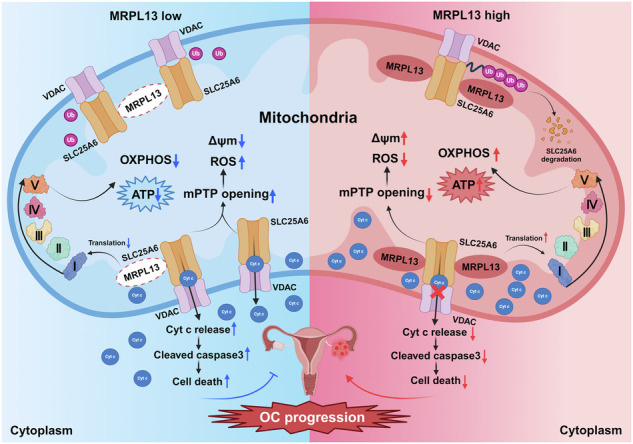

## Introduction

Ovarian cancer (OC) is one of the three most common gynecologic malignancies worldwide and ranks as the second leading cause of death among gynecologic malignancies. Annually, more than 200,000 deaths are attributed to OC, accounting for 4.8% of all cancer-related deaths in women [1]. Due to its asymptomatic nature in the early stages and the lack of effective screening methods, 70 to 80% of OC patients are diagnosed at advanced stages, characterized by extensive peritoneal metastasis. The 5-year survival rate for advanced OC is only 30% [2]. Primary debulking surgery combined with platinum-based chemotherapy remains the standard treatment for OC [3]. However, most patients experience recurrence, metastasis, and chemoresistance, making OC a fatal gynecologic malignancy [4]. As precision medicine advances in OC management, elucidating the key molecular mechanisms underlying OC initiation and progression, identifying effective biomarkers for tumor diagnosis, and developing innovative therapeutic strategies are critical to improving patient outcomes.

Mitochondria serve as the powerhouse of cellular energy metabolism and act as central coordinators of cell death signaling pathways [5]. Alterations in mitochondrial function and morphology impact tumorigenesis and progression by regulating energy metabolism, biosynthesis, redox homeostasis, transcriptional regulation, and cell death [6]. Numerous studies have highlighted the pivotal role of mitochondria in OC progression [7, 8]. Metabolic reprogramming is a hallmark of malignant transformation in tumors [9]. OC cells are highly dependent on mitochondrial oxidative phosphorylation (OXPHOS) [10, 11], which is essential for meeting the energy demands and biosynthetic needs of excessive proliferation, invasion, and metastasis. Furthermore, dysfunction of mitochondrial-associated proteins is frequently observed in OC cells, leading to abnormal energy metabolism, mitochondrial dynamics imbalance, and dysregulated programmed cell death [12, 13]. The regulation of mitochondrial-related gene expression plays a critical role in tumor initiation, progression, and therapy resistance, underscoring its potential value in tumor prognosis and therapy.

Mitochondrial ribosomal proteins (MRPs) are encoded by nuclear genes and predominantly localized in mitochondria [14]. MRPs participate in the translation of OXPHOS complex subunits encoded by mitochondrial DNA, contributing to mitochondrial protein synthesis and playing essential roles in maintaining mitochondrial homeostasis, energy metabolism, and cell death regulation [15, 16]. Aberrant expression of MRPs has been reported in various cancers and is closely associated with tumorigenesis and progression [17]. Mitochondrial ribosomal protein L13 (MRPL13), a member of the MRP family, is located on chromosome 8q24.12 and encodes the 39S large subunit of the mitochondrial ribosome. Recent studies have revealed that MRPL13 is abnormally expressed in several malignancies, such as breast cancer [18], gastric cancer [19], liver cancer [20], and lung cancer [21], and plays a significant role in driving malignant progression. However, the specific role and molecular mechanisms of MRPL13 in OC development and progression remain unclear.

Solute carrier family 25 member 6 (SLC25A6), also known as adenine nucleotide translocase 3 (ANT3), is a transport protein localized on the inner mitochondrial membrane (IMM). It participates in the exchange of mitochondrial ADP/ATP [22], plays a crucial role in the formation of mitochondrial permeability transition pore (mPTP), affects cellular energy metabolism, regulates mitochondrial function, modulates mitophagy, and induces cell death [23, 24]. The mPTP is a non-selective channel assembled at the interface of the inner and outer mitochondrial membranes, permitting diffusion of low molecular weight solutes (≤1.5 kDa) [25]. While its exact composition remains controversial, the prevailing “multi-pore model” implicates F_1_F_O_ ATP synthase, ANT, and cyclophilin D (CypD) as core components, with emerging regulators including voltage-dependent anion channel (VDAC), proapoptotic Bcl-2 family members (Bax and Bak), phosphate carrier (PiC), and translocator protein (TSPO) [26–28]. The mPTP exists in two forms: low-conductance pore and high-conductance pore. The transient opening of the mPTP may play various physiological roles, including the regulation of mitochondrial bioenergetics, cellular metabolism, Ca^2+^ homeostasis, and ROS signaling. However, under pathological conditions, the persistent opening of the mPTP can lead to the collapse of mitochondrial membrane potential, mitochondrial swelling, outer membrane rupture, and the release of cytochrome c (Cyt c), ultimately triggering cell death [29]. Notably, mPTP-mediated cell death mechanisms play a significant role in the pathogenesis of various malignancies [30, 31]. Targeting the mPTP is considered a promising therapeutic strategy for cancer. Therefore, elucidating the molecular mechanisms of mPTP-mediated tumor cell death will lay the foundation for the development of novel cancer therapies.

This study aims to elucidate the pivotal role of MRPL13 in regulating mitochondrial function and the malignant progression of OC through integrated clinical sample analysis and functional experiments, both in vitro and in vivo. Mechanistically, we demonstrate that MRPL13 specifically binds to SLC25A6 and facilitates its K48-linked ubiquitination, leading to accelerated protein degradation of SLC25A6. Notably, we aim to elucidate the mechanism by which the MRPL13-SLC25A6 axis enhances mitochondrial function and promotes tumor progression in OC by inhibiting mPTP opening. These profound findings not only provide potential molecular markers for prognostic evaluation and drug resistance monitoring in OC, but more importantly, lay a theoretical foundation for the development of targeted therapeutic strategies for OC.

## Methods

### Sample collection and clinical data

This study included pathological specimens from 191 patients who underwent surgical treatment in the Department of Gynecology at Shengjing Hospital of China Medical University. The samples consisted of 114 cases of ovarian epithelial malignant tumors, 33 cases of borderline ovarian tumors, 31 cases of benign ovarian tumors, and 13 cases of normal ovarian tissues. All cases were primary ovarian epithelial tumors with complete clinicopathological information and follow-up data. None of the patients received chemotherapy, radiotherapy, or other adjuvant treatments before surgery. Ethical approval for this study was obtained from the Ethics Committee of Shengjing Hospital, China Medical University (Approval No. 2023PS1213K). All patients were informed about the study and provided written informed consent.

### Immunohistochemical (IHC) staining

Formalin-fixed and paraffin-embedded tissue sections were processed for IHC staining using an IHC kit (Maixin, KIT-9720) to detect protein expression in the tissues. After deparaffinization, antigen retrieval, and blocking of endogenous peroxidase activity, primary antibodies were incubated overnight at 4 °C. The primary antibodies used were MRPL13 (Proteintech, 16241-1-AP, 1:100) and Ki-67 (CST, 9449, 1:2000). Subsequently, HRP-conjugated secondary antibodies were incubated at room temperature for 1 h. DAB staining (Maixin, DAB-1031) was performed, followed by hematoxylin counterstaining, dehydration, and mounting. Slides were observed under a microscope. Scoring was independently performed by two experienced pathologists in a blinded manner using the 12-point scale. The IHC score was determined by evaluating both staining intensity (0–3 points: 0 = no staining, 1 = weak staining, 2 = moderate staining, 3 = strong staining) and the percentage of positively stained cells (0–4 points: 0 ≥ 5%, 1 = 6–25%, 2 = 26–50%, 3 = 51–75%, 4 ≥ 75%). The final IHC score was calculated by multiplying the staining intensity score by the percentage of positive cells score, resulting in the following categories: 0–2 (−), 3–4 (+), 5–8 (++), and 9–12 (+++). Patients with IHC scores of 0–4 were classified as the MRPL13 low expression group (−/+), while those with scores of 5–12 were classified as the MRPL13 high expression (++/+++).

### Cell culture

Human OC cell lines OVCAR-3 and ES-2, as well as the human embryonic kidney cell line HEK293T, were purchased from the Cell Bank of the Chinese Academy of Sciences (Shanghai, China). OVCAR-3, ES-2, and HEK293T cells were cultured in RPMI 1640 medium, McCoy’s 5 A medium, and DMEM medium (VivaCell, China), respectively, supplemented with 10% fetal bovine serum (Biological Industries, Israel). All cells were maintained in a humidified atmosphere with 5% CO_2_ at 37 °C. Cell lines were authenticated by short tandem repeat (STR) profiling and tested for mycoplasma contamination.

### SiRNA, lentivirus, plasmids, and transfection

Lentiviral constructs encoding MRPL13 overexpression were synthesized by HANBIO (Shanghai, China). Cells were infected with lentivirus when the confluence reached 30–40%. At 72 h post-transfection, cells were selected with 2 μg/mL puromycin to establish stable MRPL13-overexpressing cell lines. SiRNAs targeting MRPL13 and SLC25A6 were designed and synthesized by GenePharma (Shanghai, China), with target sequences listed in Supplementary Table 1. Full-length expression vectors for Flag-MRPL13, Myc-SLC25A6, HA-Ub, and their mutants were constructed by GeneChem (Shanghai, China). Transfections of siRNAs and plasmids were performed using Lipofectamine 3000 (Invitrogen, L3000015) according to the manufacturer’s protocol. Cells were harvested 48 h post-transfection for further experiments.

### RNA extraction and quantitative Real-Time PCR (qRT-PCR)

Total RNA was extracted from transfected cells using TRIzol reagent (Takara, 9109). cDNA synthesis was performed using the PrimeScript RT reagent Kit (Takara, 047 A). Real-time PCR was conducted using the Premix Ex Taq II Kit (Takara, 820 A) on the 7500 Fast Real-Time PCR system. Results were presented as mean ± SD and normalized to the internal reference gene ACTB. Gene expression levels were analyzed using the 2^−ΔΔCt^ method. Primers were synthesized by Sangon Biotech (Shanghai, China), and sequences are provided in Supplementary Table 1.

### Western blotting

Western blotting was employed to assess protein expression levels. Samples were lysed using RIPA buffer containing PMSF, and protein concentrations were measured with the BCA Protein Assay Kit (Epizyme, ZJ102). Proteins were separated via SDS-PAGE and transferred onto 0.2 μm or 0.45 μm PVDF membranes (Millipore, USA). Membranes were blocked in 5% non-fat milk for 1–2 h at room temperature, followed by overnight incubation at 4 °C with primary antibodies, including MRPL13 (Proteintech, 16241-1-AP, 1:1000), β-Actin (Proteintech, 66009-1-Ig, 1:5000), PCNA (CST, 2586, 1:1000), Bcl-2 (Proteintech, 12789-1-AP, 1:2000), Bax (CST, 5023, 1:1000), Caspase-3 (CST, 9662, 1:1000), SLC25A6 (Proteintech, 14841-1-AP, 1:1000), Flag-tag (Proteintech, 20543-1-AP, 1:4000), HA-tag (Proteintech, 51064-2-AP, 1:4000), Myc-tag (Proteintech, 16286-1-AP, 1:4000), Ubiquitin (CST, 3936, 1:1000), Cyt c (Proteintech, 10993-1-AP, 1:1000), and COXIV (Proteintech, 11242-1-AP, 1:2000). After three washes with TBST, membranes were incubated with HRP-conjugated secondary antibodies (ZSGB-BIO, ZB-2301/ZB-2305, 1:4000) at room temperature for 1 h. Protein bands were visualized using HRP chemiluminescent substrate (Millipore, USA) and imaged with the Tanon 5200 Multi System. Band intensity was quantified using ImageJ software. All experiments were performed at least three times independently to ensure reproducibility.

### Cell viability and proliferation assays

Cell viability was assessed using the Cell Counting Kit-8 (CCK-8) assay (GLPBIO, GK10001) following the manufacturer’s instructions. Transfected cells were seeded into 96-well plates at a density of 2000 cells per well. Subsequently, 10 µL of CCK-8 solution was added to each well, and the cells were incubated at 37 °C for 3 h. Optical density (OD) was measured at 450 nm at 0, 24, 48, 72, and 96 h. Cell proliferation was evaluated using the BeyoClick^TM^ EdU-594 Cell Proliferation Kit (Beyotime, C0078) according to the manufacturer’s protocol. Cells were sequentially subjected to EdU labeling, fixation, washing, and permeabilization. The click reaction solution was added to load the probe, followed by DAPI staining for nuclei. EdU-positive nuclei were observed under a microscope, and the proportion of EdU-positive nuclei was recorded.

### Xenograft tumor model in nude mice

Female BALB/cA-nu nude mice (4–6 weeks old) were purchased from Huafukang Biosciences (Beijing, China) and maintained under specific pathogen-free (SPF) conditions. All animal experiments were approved and conducted according to the guidelines of the Ethics Committee of Shengjing Hospital of China Medical University (Approval No. 2023PS1214K). Twelve mice were blindly randomized into two groups to establish subcutaneous xenograft models for evaluating tumor growth. ES-2 cells (4 × 10^6^ cells per mouse) were subcutaneously injected into the right axilla of each mouse. Tumor growth and body weight were monitored every 3 days. After 3 weeks, the mice were humanely euthanized by CO_2_ asphyxiation, and tumors were excised, weighed, and measured for volume. Tumor volume was calculated using the formula: *V* = 1/2 × *a* × *b*^2^ (*a* is the long axis and *b* is the short axis). Tumor tissues were fixed, embedded, and sectioned for IHC and hematoxylin-eosin (H&E) staining.

### Invasion and migration assays

Cell invasion was assessed by the transwell invasion assay. The assay was conducted in a 24-well plate with transwell inserts containing an 8 μm pore-size membrane (Corning Costar, USA). The upper chambers were pre-coated with matrigel (Corning Costar, 356234) diluted in serum-free medium. OC cells (4 × 10^4^ cells per well) in serum-free medium were seeded into the upper chambers, while the lower chambers were filled with 500 µL of medium containing 20% fetal bovine serum as the chemoattractant. After 48 h of incubation, the inserts were fixed, stained with crystal violet, and cells remaining on the upper side of the membrane were removed using a cotton swab. Cells that had invaded through the membrane were counted under a microscope. Cell migration ability was evaluated using the wound-healing assay. Confluent OC cells in 6-well plates were scratched using a sterile 200 μL pipette tip. The cells were then cultured in serum-free medium, and the migration area was observed and measured at 0 h and 24 h.

### Flow cytometry for apoptosis detection

Cell apoptosis was measured using the Annexin V-FITC/PI dual-staining apoptosis detection kit (KeyGen Biotech, KGA1102) and the Annexin V-APC/PI dual-staining kit (KeyGen Biotech, KGA1107). After harvesting the cells, the cell pellets were incubated with the staining solution for 30 min at room temperature in the dark. The apoptotic rate was analyzed using the flow cytometer (BD Biosciences). Data were processed using FlowJo software (version 10.8.1).

### Mitochondrial membrane potential and reactive oxygen species (ROS) level detection

Mitochondrial membrane potential (MMP) was measured using the JC-1 Mitochondrial Membrane Potential Assay Kit (Beyotime, C2006). Cells were incubated with JC-1 staining working solution for 20 min. After incubation, cells were washed with ice-cold staining buffer twice and visualized under the fluorescence microscope. The mitochondrial membrane potential was evaluated based on the fluorescence intensity ratio of JC-1 aggregates (red) to JC-1 monomers (green). ROS levels were detected using ROS Assay Kit (Beyotime, S0033). The DCFH-DA probe was diluted in serum-free medium at 1:2000 ratio and incubated with cells for 20 min. Fluorescent signals were quantified using the flow cytometer (BD Biosciences) to analyze cellular ROS levels.

### Oxygen consumption rate (OCR) measurement

The oxygen consumption rate (OCR) was measured using the Seahorse XF96 Extracellular Flux Analyzer (Seahorse Bioscience, Agilent, USA) according to the manufacturer’s instructions for the Seahorse XF Cell Mitochondrial Stress Test Kit (Agilent, 103015). OC cells were seeded into XF96 cell culture plates at a density of 1 × 10^4^ cells per well and incubated overnight at 37 °C. The medium was replaced with assay solution containing XF DMEM Medium (Agilent, 103575) supplemented with 1 mM sodium pyruvate (Agilent, 103578), 10 mM glucose (Agilent, 103577), and 2 mM glutamine (Agilent, 103579). Cells were incubated in a non-CO₂ incubator for 1 h prior to measurement. Mitochondrial stress was evaluated by automated sequential injections of 1.5 µM Oligomycin, 1.5 µM FCCP, and 0.5 µM Rotenone/Antimycin A. Data were analyzed using Seahorse Wave Controller software to assess OCR.

### ATP level

Cellular ATP content was quantified using the ATP Assay Kit (Beyotime, S0026) following the manufacturer’s protocol. Cells were lysed, and 100 µL of working solution was added to each well and incubated at room temperature for 5 min. Subsequently, 20 µL of cell lysate was added to each well, and relative light units (RLU) were measured using a luminometer to quantify ATP levels.

### MPTP opening detection

The degree of mPTP opening was assessed using the mPTP Assay Kit (Beyotime, C2009). Cells were incubated with Calcein AM staining solution and fluorescence quenching working solution containing CoCl_2_ for 30 min at 37 °C in the dark. After replacing the medium, cells were incubated in fresh medium in the dark for an additional 30 min. Detection buffer was added, and the fluorescence intensity of Calcein (green fluorescence) was observed under a fluorescence microscope. The intensity of green fluorescence was used as an indicator of mPTP opening.

### MitoTracker staining

OC cells were seeded in confocal culture dishes and stained with 200 nM MitoTracker Red CMXRos (Beyotime, C1035) staining solution, prepared according to the manufacturer’s instructions. The cells were incubated at 37 °C in the dark for 30 min. After incubation, nuclei were counterstained with Hoechst 33342 staining solution (Beyotime, C1027). The medium was replaced with pre-warmed fresh medium, and cells were observed under a laser scanning confocal microscope (Zeiss, LSM880, Germany). Mitochondrial morphology was analyzed using ImageJ Mitochondria Analyzer.

### Transmission electron microscopy (TEM)

OC cells were fixed overnight at 4 °C with 2.5% glutaraldehyde, followed by fixation with 1% osmium tetroxide at 4 °C for 1 h. Cells were dehydrated through a graded ethanol series, infiltrated with acetone, embedded in epoxy resin, and sectioned into ultrathin slices (50 nm). The sections were stained with uranyl acetate and lead citrate, and the ultrastructure of mitochondria was observed using a JEM-1400Flash transmission electron microscope (JEOL, Japan).

### Co-Immunoprecipitation (Co-IP)

OC cells were lysed using IP lysis buffer (Beyotime, P0013) containing protease and phosphatase inhibitors. The lysates were centrifuged, and 1% of the supernatant was retained as input, while the remaining supernatant was transferred to a fresh tube and incubated overnight at 4 °C with 2 μg of the respective IP antibody per sample. The next day, Protein A/G magnetic beads (MCE, HY-K0202) were pre-washed with IP lysis buffer and added to the antibody-protein mixture for incubation at 4 °C for 4–6 h. The beads were washed three times with IP lysis buffer, and bound proteins were eluted by adding loading buffer followed by heating. The eluted samples were subjected to western blotting analysis. Antibodies used in Co-IP included MRPL13 (Proteintech, 16241-1-AP), SLC25A6 (Proteintech, 14841-1-AP), Flag-tag (Proteintech, 66008-4-Ig), HA-tag (Proteintech, 66006-2-Ig), Myc-tag (Proteintech, 60003-2-Ig), Rabbit IgG (Beyotime, A7016), and Mouse IgG (Beyotime, A7028).

### LC-MS/MS analysis

Protein samples collected from IP experiments were separated using SDS-PAGE gel electrophoresis and stained with a Coomassie Brilliant Blue staining kit (Solarbio, P1305). After staining and washing, the gel bands were excised into small strips and submitted to the BGI Protein Research Center (Beijing, China) for liquid chromatography-tandem mass spectrometry (LC-MS/MS) analysis. This analysis was performed to identify proteins interacting with MRPL13.

### Immunofluorescence (IF) staining

OC cells were seeded in confocal culture dishes and fixed with 4% paraformaldehyde. After PBS washing, the cells were permeabilized with Triton X-100 (Beyotime, P0096) for 20 min. Cells were then blocked with 10% goat serum for 30 min and incubated overnight at 4 °C with primary antibodies. The next day, after three PBST washes, the cells were incubated at 37 °C with fluorescence-conjugated secondary antibodies for 1 h. Following another set of PBST washes, the nuclei were counterstained with DAPI (Solarbio, C0065) for 10 min. The cells were observed under a laser scanning confocal microscope (Zeiss, LSM880, Germany).

### Molecular docking

The crystal structure of MRPL13 (PDB ID: 7QI4) was obtained from the RCSB Protein Data Bank, and the crystal structure of SLC25A6 was predicted using AlphaFold2. Protein crystals were processed using the Protein Preparation Wizard module of Schrödinger software by performing the following operations: protein preprocess, regenerate states of native ligand, H-bond assignment optimization, protein energy minimization, and remove waters. Protein–protein interaction simulations were performed using the prepared protein structures. Different chains in the interaction complex were highlighted with distinct colors, surfaces were added, and 3D structures were visualized. The Protein Interaction Analysis module was used to determine the specific binding regions between MRPL13 and SLC25A6.

### Mitochondrial isolation

Mitochondria from OC cells were isolated using the Cell Mitochondria Isolation Kit (Beyotime, C3601), and mitochondrial-free cytoplasmic proteins were obtained. Cells were collected and incubated with mitochondrial isolation reagent containing PMSF on ice for 15 min. The mixture was homogenized 20 times using a glass homogenizer and centrifuged at 600 × *g* for 10 min at 4 °C to collect the supernatant. This supernatant was centrifuged again at 11,000 × *g* for 10 min at 4 °C, and the resulting pellet was the isolated mitochondria. Mitochondria were lysed with mitochondrial lysis buffer to obtain mitochondrial proteins. The supernatant was collected and centrifuged at 12,000 × *g* for 10 min at 4 °C. The supernatant obtained again was mitochondrial-free cytoplasmic proteins. Protein levels of cytochrome c in the cytoplasm were analyzed using western blotting.

### Bioinformatics analysis

The GEPIA database (http://gepia.cancer-pku.cn/) was used to analyze the differences in MRPL13 mRNA levels between OC and normal ovarian tissues. The UALCAN database (ualcan.path.uab.edu) was utilized to visualize MRPL13 protein expression levels in OC compared to normal ovarian tissues. Datasets GSE26712, GSE54388, GSE18520, GSE27651, and GSE36668 were downloaded from the GEO database (www.ncbi.nlm.nih.gov/geo/) for MRPL13 mRNA expression analysis. The Kaplan-Meier plotter database (http://kmplot.com) was employed to evaluate the prognostic impact of MRPL13 on OC. Utilizing the “co-expression” module of the cBioPortal database (https://www.cbioportal.org/), a list of genes co-expressed with MRPL13 was obtained from the ovarian serous cystadenocarcinoma dataset (TCGA, Nature 2011). By setting a threshold of Spearman correlation coefficient > 0.30 and *p* < 0.05, 733 genes co-expressed with MRPL13 were identified. Subsequently, the DAVID database (https://david.ncifcrf.gov) was employed to perform GO and KEGG functional enrichment analyses on these co-expressed genes. Visualization of the enrichment results was achieved using the R language packages ggplot2 and GOplot. The normalized gene expression matrix for TCGA-OV was obtained from the UCSC Xena database (http://xena.ucsc.edu/). The c2.cp.kegg.v6.2.symbols.gmt gene set was downloaded from the gene set enrichment analysis (GSEA) database (http://software.broadinstitute.org/gsea/index.jsp). Based on the expression level of MRPL13, the samples were sorted in descending order, with the top 50% designated as the MRPL13 high-expression group and the bottom 50% as the MRPL13 low-expression group. Subsequently, GSEA software (version 4.3.3) was utilized to perform gene set enrichment analysis.

### Statistical analysis

All experiments were conducted at least three times independently. Statistical analyses and data visualization were performed using SPSS statistical software (version 26.0) and GraphPad Prism (version 8.0.1). The chi-square test was used to assess the relationship between gene expression and clinicopathological features of patients. Kaplan–Meier analysis was used for survival analysis and compared by the Log-rank test. Cox regression models were used for univariate and multivariate analyses. Normality and homogeneity of variance tests were evaluated first. For comparison of quantitative data between two or multiple unpaired groups, Student’s *t*-test and one-way ANOVA were applied, respectively. Data are presented as mean ± standard deviation (SD). A two-tailed *P* value of <0.05 was considered statistically significant (*, *P* < 0.05; **, *P* < 0.01; ***, *P* < 0.001).

## Results

### MRPL13 is an oncogene associated with OC progression and poor clinical prognosis

**Fig. 1:**
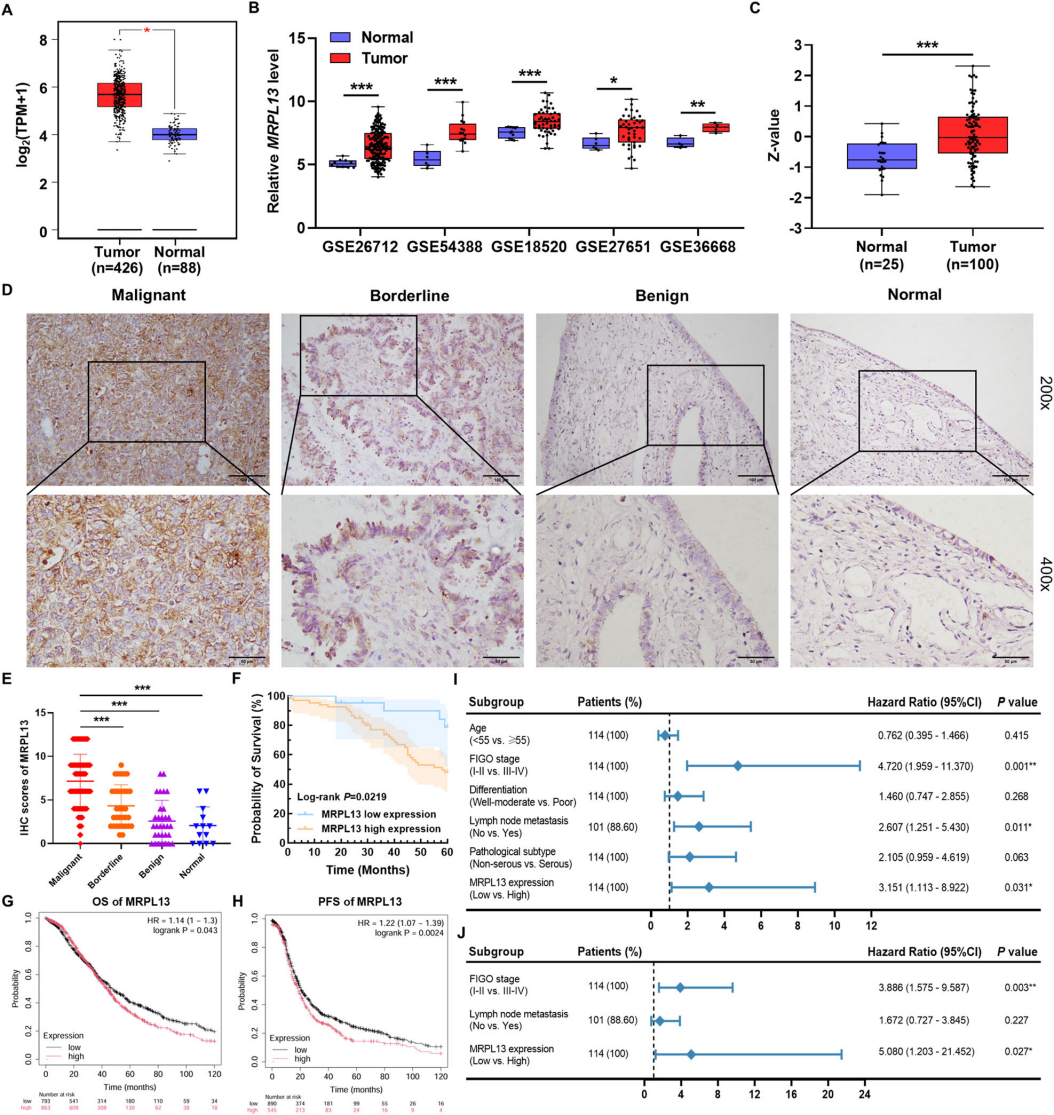
MRPL13 is an oncogene associated with OC progression and poor clinical prognosis. **A** Relative MRPL13 mRNA expression in the GEPIA database. Box plots show MRPL13 mRNA expression in OC (*n* = 426) and the corresponding normal tissues (*n* = 88). Axis unit is log2(TPM + 1). **B** Relative MRPL13 mRNA expression in OC and normal tissues based on data from the GEO database (GSE26712, GSE54388, GSE18520, GSE27651 and GSE36668). **C** Relative MRPL13 protein expression in OC (*n* = 100) and normal tissues (*n* = 25) based on data from the UALCAN database. **D** Representative images of MRPL13 IHC staining in ovarian malignant tumors (*n* = 114), ovarian borderline tumors (*n* = 33), ovarian benign tumors (*n* = 31) and ovarian normal tissues (*n* = 13). Scale bar, 100 μm (×200, upper) and 50 μm (×400, lower). **E** Scatter plot of MRPL13 IHC staining scores in different ovarian tissues. **F** Kaplan–Meier analysis of the OS of patients with OC in the Shengjing hospital cohort based on MRPL13 expression evaluated in. **G**, **H** Kaplan–Meier analysis of the OS and PFS of patients with OC in Kaplan–Meier plotter database based on MRPL13 expression. **I**, **J** Forest plots of univariate and multivariate Cox regression analyses for various clinicopathological parameters in OC patients. *, *P* < 0.05; **, *P* < 0.01; ***, *P* < 0.001.

We first visualized MRPL13 mRNA expression using the GEPIA database, integrating data from The Cancer Genome Atlas (TCGA, *n* = 426) and the Genotype-Tissue Expression (GTEx, *n* = 88) database. The transcriptional levels of MRPL13 were significantly higher in OC tissues compared to normal ovarian tissues (Fig. 1A). To further confirm this finding, we analyzed multiple public datasets from the Gene Expression Omnibus (GEO), including GSE26712, GSE54388, GSE18520, GSE27651, and GSE36668. All datasets consistently demonstrated the upregulation of MRPL13 in OC (Fig. 1B). Next, we evaluated MRPL13 protein expression in OC using the UALCAN, which included 100 OC tissues and 25 normal tissues in the CPTAC-OC dataset. The results showed that MRPL13 protein levels were elevated in OC (Fig. 1C).

To validate the abnormal expression of MRPL13 in OC, we performed IHC analysis on samples from the Shengjing Hospital OC cohort. The results indicated that both the positive expression rate and high positive expression rate of MRPL13 were significantly higher in ovarian epithelial malignant tumors (93.86% and 73.68%, respectively) compared to borderline ovarian tumors (69.70% and 39.39%, respectively), benign ovarian tumors (41.94% and 19.35%, respectively), and normal ovarian tissues (30.77% and 15.38%, respectively). MRPL13 expression increased progressively with the progression of malignancy in ovarian tissues (Fig. 1D, E and Table 1). A total of 114 samples of OC were divided into MRPL13 high-expression group (++/+++) and MRPL13 low-expression group (−/+) based on IHC staining scores. We further explored the association between MRPL13 expression and clinicopathological parameters in 114 OC patients. Clinical correlation analysis revealed that high positive expression of MRPL13 was closely associated with the International Federation of Gynecology and Obstetrics (FIGO) stage of OC patients, suggesting that MRPL13 overexpression contributes to OC progression. Detailed associations between MRPL13 expression and clinicopathological parameters are presented in Table 2.

**Table 1:** Expression of MRPL13 in different types of ovarian tissue.

Groups	Cases	Low		High		Positive rate (%)	High positive rate (%)
		-	+	++	+++		
Malignant	114	7	23	40	44	93.86^a,b^	73.68^c,d^
Borderline	33	10	10	12	1	69.70^e,f^	39.39^g,h^
Benign	31	18	7	6	0	41.94	19.35
Normal	13	9	2	2	0	30.77	15.38

^a^Malignant vs. benign (***, *P* < 0.001).

^b^Malignant vs. normal (***, *P* < 0.001).

^c^Malignant vs. benign (***, *P* < 0.001).

^d^Malignant vs. normal (***, *P* < 0.001).

^e^Borderline vs. benign (*, *P* < 0.05).

^f^Borderline vs. normal (*, *P* < 0.05).

^g^Borderline vs. benign (*P* = 0.080).

^h^Borderline vs. normal (*P* = 0.224).

**Table 2:** Relationship between MRPL13 expression and clinicopathological parameters of ovarian epithelial malignant tumors.

Groups	Cases	Low		High		Positive rate (%)	*P* value	High positive rate (%)	*P* value
		-	+	++	+++				
**Age**							0.439		0.754
<55	58	5	11	17	25	91.38		72.41	
≥55	56	2	12	23	19	96.43		75.00	
**FIGO stage**							0.431		0.025*
I-II	45	4	13	15	13	91.11		62.22	
III-IV	69	3	10	25	31	95.65		81.16	
**Differentiation**							0.714		0.336
Well-moderate	56	4	13	17	22	92.86		69.64	
Poor	58	3	10	23	22	94.83		77.59	
**Lymphatic metastasis**							0.350		0.815
No	72	3	16	26	27	95.83		73.61	
Yes	29	3	4	11	11	89.66		75.86	
Unknown	13	1	3	3	6	92.31		69.23	
**Pathological type**							0.723		0.239
Serous	71	5	13	30	23	92.96		74.65	
Mucinous	10	0	4	2	4	100.00		60.00	
Endometrioid	20	2	5	5	8	90.00		65.00	
Clear cell carcinoma	13	0	1	3	9	100.00		92.31	

Comprehensive follow-up was conducted on the 114 patients with OC enrolled in the study (up to March 31, 2023). Kaplan-Meier analysis revealed that high expression of MRPL13 correlated with poor prognosis in OC patients, with significantly shorter overall survival (OS) observed in the high MRPL13 expression group compared to the low expression group (Fig. 1F). Further survival analysis using the Kaplan-Meier plotter online database corroborated these findings, showing that patients with high MRPL13 expression had worse OS and progression-free survival (PFS) (Fig. 1G, H). Cox regression analysis was performed to identify prognostic risk factors associated with survival outcomes in OC patients. Univariate analysis indicated that MRPL13 expression, FIGO stage, and lymph node metastasis were significantly correlated with survival prognosis (Fig. 1I). Multivariate analysis confirmed that MRPL13 expression and FIGO stage were independent prognostic risk factors for OC patient survival (Fig. 1J). In summary, these results demonstrate that MRPL13 upregulation is strongly associated with OC progression and poor prognosis, underscoring its potential as a biomarker and therapeutic target.

To further validate our findings, we performed qRT-PCR to assess MRPL13 mRNA expression levels in different ovarian tissues. Consistent with the IHC and bioinformatics analysis results, MRPL13 mRNA levels were significantly higher in ovarian epithelial malignant tumors than in benign ovarian tumors and normal ovarian tissues (Supplementary Fig. 1).

### MRPL13 promotes malignant progression of OC cells in vitro and in vivo

**Fig. 2:**
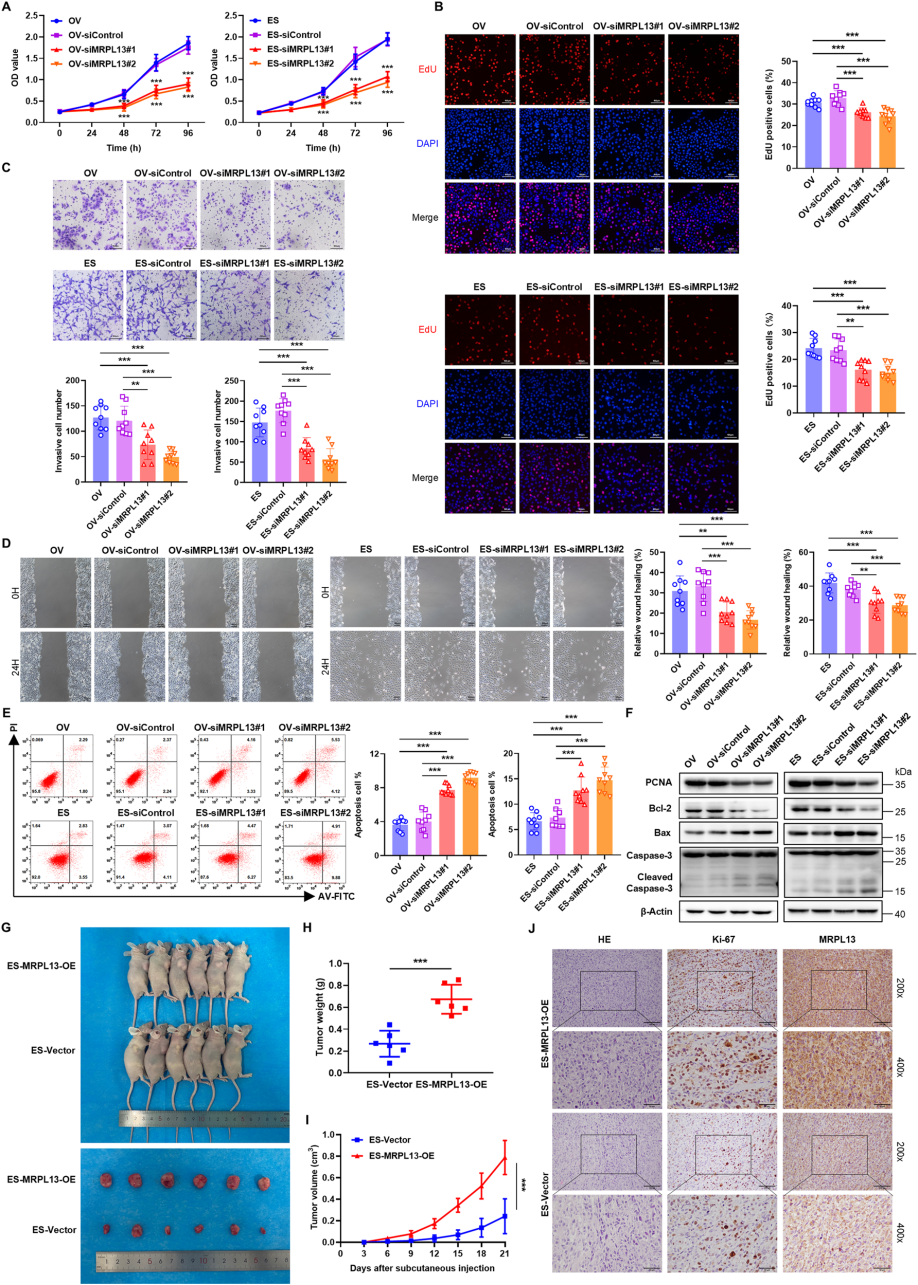
MRPL13 promotes malignant progression of OC cells in vitro and in vivo. **A** CCK-8 assays were utilized to evaluate the cell viability in OVCAR-3 and ES-2 cells with MRPL13 knockdown. *: siControl vs siMRPL13. **B** EdU assays were conducted to assess cell proliferation in OVCAR-3 and ES-2 cells with MRPL13 knockdown. DAPI (blue) staining was performed to indicate total cells, while EdU (red) incorporation indicated cells with active DNA replication. **C** Transwell assays were conducted to assess cell invasion ability in OVCAR-3 and ES-2 cells with MRPL13 knockdown. **D** Wound healing assays were used to evaluate cell migration ability in OVCAR-3 and ES-2 cells with MRPL13 knockdown. **E** Cell apoptosis of OVCAR-3 and ES-2 cells in the MRPL13 knockdown groups was validated by flow cytometry. **F** Protein expression level of PCNA, Bcl-2, Bax, Caspase-3 and Cleaved Caspase-3 were monitored by western blot for lysates from OVCAR-3 and ES-2 cells with MRPL13 knockdown. β-Actin was used as an internal control. All assays were performed in three independent experiments. **G** Subcutaneous xenograft tumor model of OC was established in BALB/c nude mice with MRPL13 stable overexpression ES-2 cells. **H** The weights of subcutaneous xenograft tumors were measured after euthanized. **I** Tumor volumes were measured at three-day intervals following the subcutaneous injection to generate tumor growth curve. **J** Representative images of Hematoxylin-eosin (H&E) staining and IHC staining on the paraffin sections of xenograft tumors to visualize the expression of Ki-67 and MRPL13. Scale bar, 100 μm (×200, upper) and 50 μm (×400, lower). Data are presented as mean ± SD. **, *P* < 0.01; ***, *P* < 0.001.

To investigate the role of MRPL13 in OC progression, we first established MRPL13 knockdown cell lines in OVCAR-3 and ES-2 cells using two distinct siRNA sequences, as well as stable MRPL13-overexpressing cell lines using the lentiviral delivery system. The knockdown and overexpression of MRPL13 were confirmed at both the protein and mRNA levels (Supplementary Fig. 2A–D). The CCK-8 assay revealed that MRPL13 knockdown significantly suppressed the viability of OVCAR-3 and ES-2 cells (Fig. 2A), while MRPL13 overexpression enhanced OC cell viability, particularly at 72 and 96 h (Supplementary Fig. 3A). Next, we assessed the proliferation rate of OC cells using an EdU incorporation assay. The results showed that MRPL13 knockdown reduced the proportion of EdU-positive cells compared to control groups, indicating the significant inhibition of OC cell proliferation (Fig. 2B). Conversely, MRPL13 overexpression markedly increased the proportion of EdU-positive cells, demonstrating enhanced cell proliferation (Supplementary Fig. 3B).

To analyze the regulatory role of MRPL13 in OC metastasis, we evaluated the invasion and migration abilities of OC cells using transwell and wound healing assays, respectively. Knockdown of MRPL13 significantly reduced the invasion and migration abilities of OC cells compared to the control group (Fig. 2C, D). In contrast, MRPL13 overexpression enhanced the invasive and migratory capacities of OC cells (Supplementary Fig. 3C, D).

Furthermore, we assessed the effect of MRPL13 on cell apoptosis using flow cytometry. By Annexin V-FITC/PI double staining, we observed that the apoptosis rate of OC cells was significantly increased after inhibition of MRPL13 expression (Fig. 2E). Conversely, Annexin V-APC/PI staining revealed that MRPL13 overexpression significantly decreased the overall apoptosis rate (Supplementary Fig. 3E). We further examined the expression of key proteins involved in the regulation of apoptosis. MRPL13 knockdown reduced the expression of the anti-apoptotic protein Bcl-2, increased the expression of the pro-apoptotic protein Bax, and induced the cleavage of caspase-3, resulting in elevated levels of cleaved caspase-3 (Fig. 2F and Supplementary Fig. 3F). Conversely, MRPL13 overexpression produced the opposite effects, suppressing apoptosis (Supplementary Fig. 3G, H). Collectively, these results clearly demonstrate the oncogenic role of MRPL13 in OC by promoting cell invasion and migration, while inhibiting apoptosis.

Subsequently, we extended our in vivo studies by developing a subcutaneous xenograft tumor model in nude mice to further evaluate the role of MRPL13 in OC cell proliferation. ES-MRPL13-OE cells and ES-Vector cells were injected subcutaneously into nude mice (*n* = 6). MRPL13 overexpression significantly promoted tumor growth and increased tumorigenic capacity (Fig. 2G). Tumor weight and volume were significantly higher in the MRPL13-overexpression group compared to the control group (Fig. 2H, I). Additionally, Ki-67 IHC staining of tumor tissues revealed higher Ki-67 intensity and area in the MRPL13-overexpression group (Fig. 2J), indicating that MRPL13 accelerates OC cell division and acts as a critical regulator of OC cell proliferation. These results confirm that MRPL13 promotes OC cell proliferation and tumor growth both in vitro and in vivo.

### MRPL13 improves mitochondrial function in OC cells

**Fig. 3:**
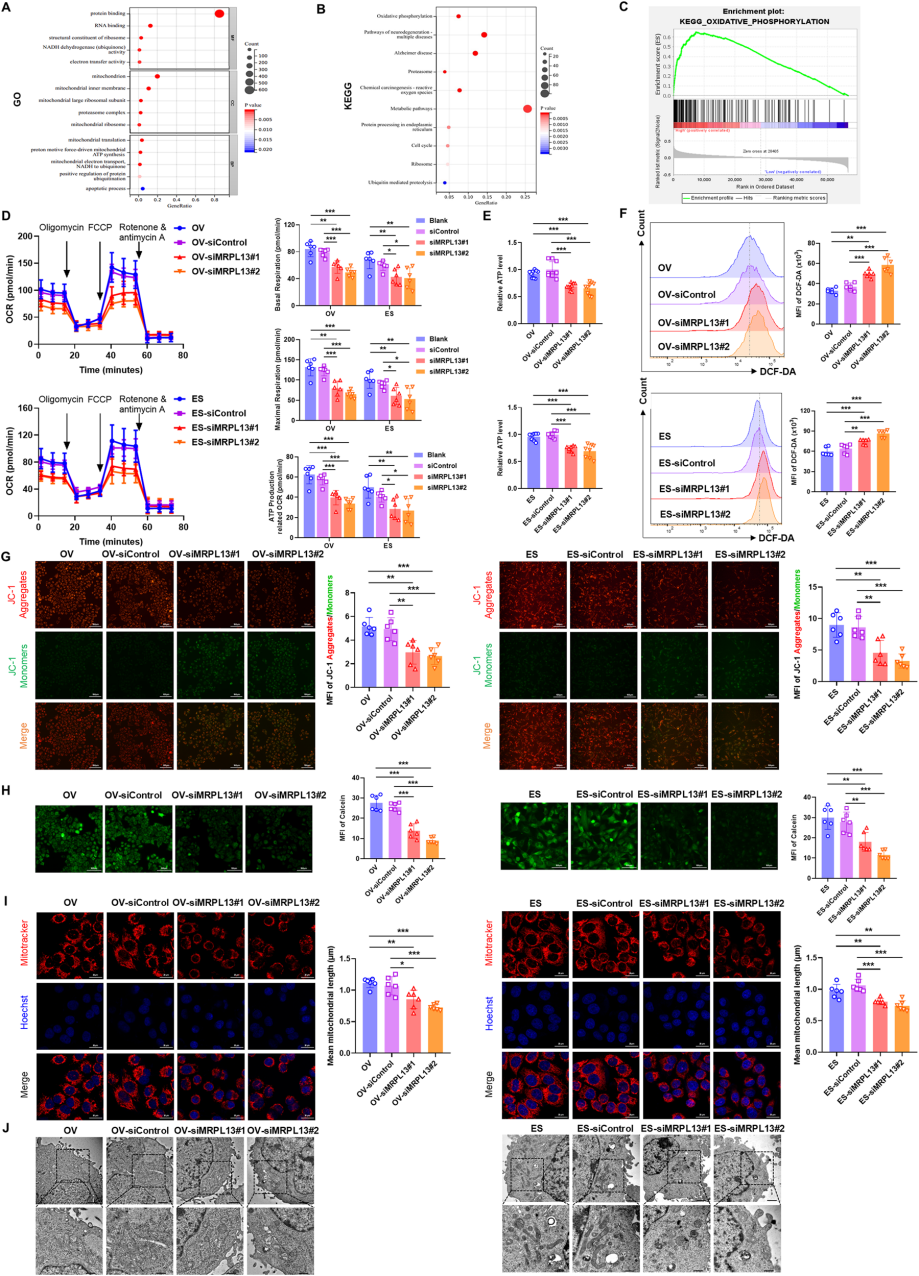
MRPL13 improves mitochondrial function in OC cells. **A**, **B** Bubble plots of Gene Ontology (GO) and Kyoto Encyclopedia of Genes and Genomes (KEGG) analysis results for the co-expressed genes of MRPL13 based on David database. **C** Results of GSEA comparing the enrichment of the oxidative phosphorylation pathway between patients with high MRPL13 expression and low MRPL13 expression in TCGA-OV cohort. **D** OCR was measured in OVCAR-3 and ES-2 cells with MRPL13 knockdown using Seahorse XF96 Extracellular Flux Analyzer. Basal respiration, maximal respiration and ATP production related OCR were quantitatively calculated. **E** Relative ATP level was recorded in OVCAR-3 and ES-2 cells with MRPL13 knockdown by ATP assay kit. **F** The mean fluorescence intensity (MFI) of DCF-DA was quantitatively calculated by flow cytometry to assess the ROS level in OVCAR-3 and ES-2 cells with MRPL13 knockdown. **G** Mitochondrial membrane potential in OVCAR-3 and ES-2 cells with MRPL13 knockdown was determined by quantitative analysis of the MFI of JC-1 aggregates (red)/monomers (green). **H** The degree of mPTP opening in OVCAR-3 and ES-2 cells with MRPL13 knockdown was detected by quantitative analysis of the MFI of Calcein AM fluorescent probe. **I** Fluorescence staining (Mito-Tracker Red CMXRos, red; Hoechst, blue) showing the mitochondrial morphology in OVCAR-3 and ES-2 cells with MRPL13 knockdown. The mitochondrial length was quantitatively analyzed by ImageJ Mitochondria Analyzer. **J** Representative images of TEM showing the mitochondrial morphology in OVCAR-3 and ES-2 cells with MRPL13 knockdown. All assays were performed in three independent experiments. Data are presented as mean ± SD. *, *P* < 0.05; **, *P* < 0.01; ***, *P* < 0.001.

MRPL13 encodes the 39S subunit of the mitochondrial ribosome, playing a crucial role in mitochondrial translation and protein synthesis. To investigate the potential mechanism by which MRPL13 drives OC progression, we used the cBioPortal database to screen out 733 co-expressed genes with MRPL13 (Spearman correlation coefficient >0.30), and then performed functional and pathway enrichment analyses of these co-expressed genes by DAVID database. GO analysis revealed that MRPL13 is primarily involved in mitochondrial translation, proton-driven mitochondrial ATP synthesis, mitochondrial electron transport, regulation of protein ubiquitination and cell apoptotic process (Fig. 3A). KEGG analysis further associated MRPL13 with OXPHOS, metabolic pathways, ROS, and the ubiquitin-proteasome pathway (Fig. 3B). GSEA analysis confirmed a positive correlation between MRPL13 expression and OXPHOS activity (Fig. 3C), highlighting the critical role of MRPL13 in regulating mitochondrial activity in OC cells.

Next, we further investigated the impact of MRPL13 on mitochondrial function. By measuring OCR levels using the Seahorse XFe 96 Extracellular Flux Analyzer, we assessed the effect of MRPL13 on the process of OXPHOS. The results indicated that MRPL13 knockdown significantly reduced OCR, including basal respiration, maximal respiration, and ATP production-linked OCR (Fig. 3D). ATP content in OC cells was also markedly decreased following MRPL13 knockdown (Fig. 3E). Flow cytometry was used to detect the mean fluorescence intensity (MFI) of DCF-DA to evaluate ROS levels, and the results showed that ROS production was significantly increased in MRPL13 knockdown cells (Fig. 3F). JC-1 staining demonstrated that MRPL13 knockdown induced mitochondrial depolarization, leading to the conversion of JC-1 from aggregates (red fluorescence) to monomers (green fluorescence), and decrease in mitochondrial membrane potential (Fig. 3G). The decrease in mitochondrial membrane potential is an early hallmark event of cell apoptosis. Sustained mPTP opening disrupts mitochondrial permeability, leading to mitochondrial membrane potential loss, increased ROS production, ATP depletion, and ultimately cell death [29]. Calcein AM staining indicated that MRPL13 knockdown significantly increased mPTP opening in OC cells (Fig. 3H). These findings suggest that MRPL13 knockdown impairs mitochondrial membrane potential and induces apoptosis by promoting mPTP opening.

Additionally, we further investigated the impact of MRPL13 knockdown on mitochondrial morphology. Confocal microscopy images showed that mitochondria became shorter and fragmented in MRPL13 knockdown cells (Fig. 3I). TEM analysis demonstrated that the inhibition of MRPL13 expression led to mitochondrial structural damage, characterized by mitochondrial swelling, disruption of cristae structure, and shortened length (Fig. 3J). Together, these results emphasize the critical role of MRPL13 in maintaining mitochondrial function and morphology, implicating its involvement in OC progression.

### MRPL13 specifically interacts with SLC25A6

**Fig. 4:**
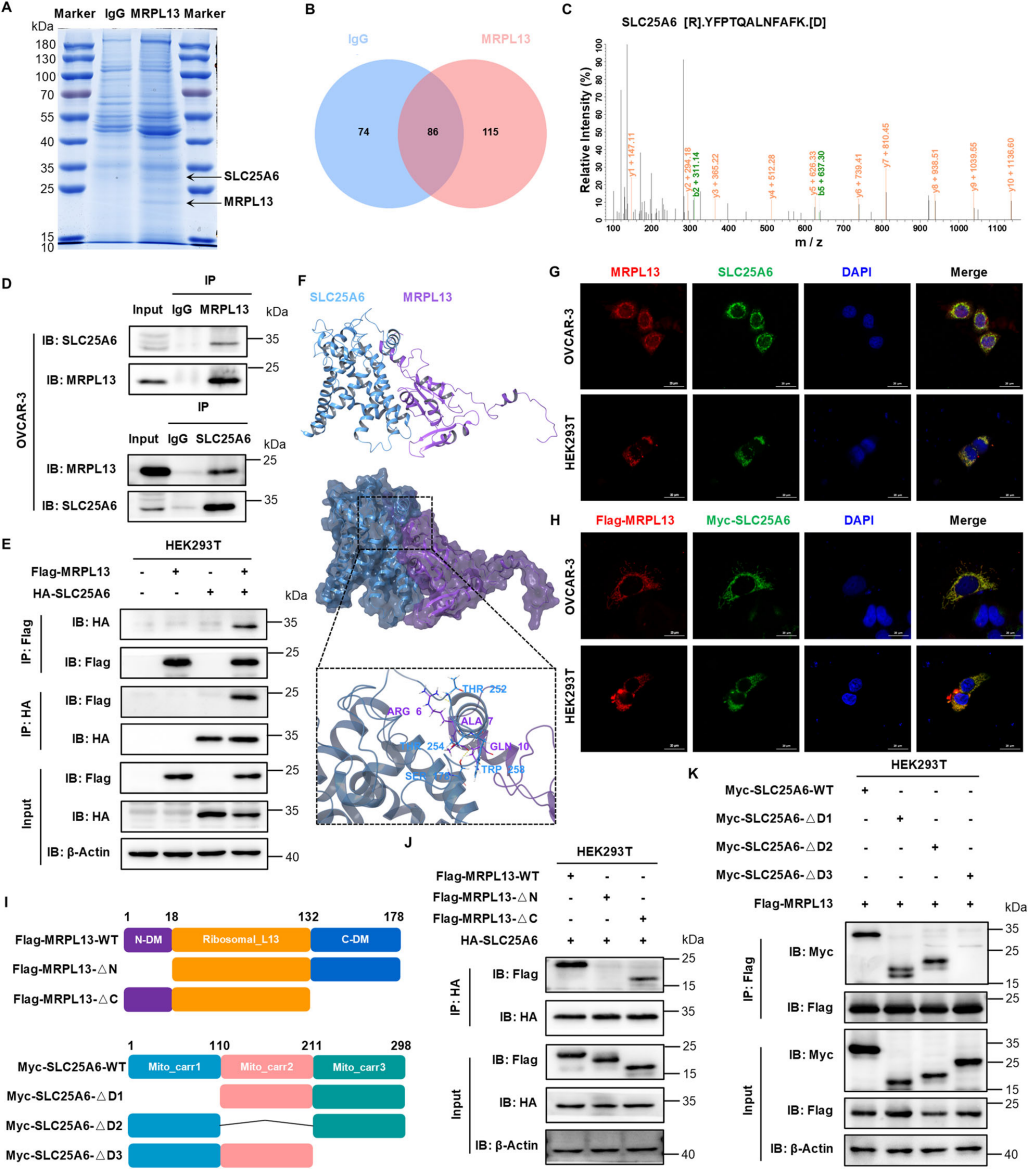
MRPL13 specifically interacts with SLC25A6. **A** Proteins that interacted with MRPL13 were identified by combining Co-IP and mass spectrometry. The images of coomassie brilliant blue staining were displayed, and the locations of target proteins were marked with black arrows. **B** Venn diagram shows the number of proteins interacting with MRPL13 or IgG in mass spectrometry result. **C** Secondary mass spectrum of SLC25A6 peptide interacting with MRPL13 from the results of mass spectrometry. **D** Binding between endogenous MRPL13 and SLC25A6. Lysates of OVCAR-3 cells were immunoprecipitated in the presence of control IgG and with anti-MRPL13 or anti-SLC25A6 antibodies. Immunoblotting was performed to detect the MRPL13 and SLC25A6 precipitates using anti-SLC25A6 and anti-MRPL13 antibodies, respectively. **E** Binding between exogenous MRPL13 and SLC25A6. HEK293T cells were transiently transfected with plasmids encoding either Flag-MRPL13 or HA-SLC25A6 alone or in combination. Cell lysates were immunoprecipitated in the presence of anti-Flag or anti-HA antibodies. Immunoblotting was performed using anti-HA and anti-Flag antibodies, respectively. **F** Molecular docking of the 3D structure revealed the tight interaction between MRPL13 (purple) and SLC25A6 (blue). **G** Visualization of MRPL13 and SLC25A6 endogenous interaction. OVCAR-3 and HEK293T cells were analyzed by IF staining for detecting the localization of MRPL13 (red) and SLC25A6 (green). DAPI was used as a nuclear stain (blue). **H** Visualization of MRPL13 and SLC25A6 exogenous interaction. OVCAR-3 and HEK293T cells were co-transfected with plasmids encoding Flag-MRPL13 and Myc-SLC25A6 and analyzed by IF staining for detecting the localization of Flag (red) and Myc (green). DAPI was used as a nuclear stain (blue). **I** Schematic representation of the domain of full-length (FL) MRPL13 or SLC25A6 and different truncation mutants. **J** Lysates from HEK293T cells were transiently transfected with HA-SLC25A6 and FLAG-tagged FL or truncated MRPL13 constructs and immunoprecipitated with anti-HA antibody, followed by immunoblotting with anti-Flag and anti-HA antibodies. **K** Lysates from HEK293T cells were transiently transfected with Flag-MRPL13 and Myc-tagged FL or truncated SLC25A6 constructs and immunoprecipitated with anti-Flag antibody, followed by immunoblotting with anti-Myc and anti-Flag antibodies. All assays were performed in three independent experiments.

To explore the molecular mechanism by which MRPL13 promotes OC malignancy, we used Co-IP combined with LC-MS/MS to identify potential interacting proteins of MRPL13. MS analysis identified a total of 201 proteins in the MRPL13 group compared to 160 proteins in the IgG control group, with 115 proteins uniquely detected in the MRPL13 group which represent potential interactors of MRPL13 (Fig. 4A–C). SLC25A6, a key component of the pro-apoptotic mPTP, was a prominent candidate. SLC25A6 plays a significant role in maintaining mitochondrial membrane permeability, regulating mitochondrial function, and inducing cell death, thereby influencing tumor progression [24]. To validate the proteomic findings, endogenous Co-IP using OVCAR-3 cell lysates confirmed the interaction between MRPL13 and SLC25A6 (Fig. 4D). Exogenous Co-IP in HEK293T cell lysates overexpressing MRPL13 and SLC25A6 further corroborated this interaction (Fig. 4E). Molecular docking analysis supported the formation of the stable MRPL13-SLC25A6 complex based on their protein crystal structures. The docking results showed that MRPL13 residues Arg6, Ala7, and Gln10 formed hydrogen bonds with SLC25A6 residues Thr252, Ser178, and Trp258, respectively, providing a molecular understanding of their interaction (Fig. 4F). Furthermore, IF analysis demonstrated the endogenous and exogenous co-localization of MRPL13 and SLC25A6 (Fig. 4G, H). To determine the specific regions mediating the MRPL13-SLC25A6 interaction, we constructed recombinant plasmids encoding truncated forms of Flag-MRPL13 and Myc-SLC25A6 (Fig. 4I). Co-IP experiments revealed that only the truncated forms of MRPL13 that retained the N-terminal region were able to bind to SLC25A6, indicating that the N-terminus of MRPL13 is crucial for their interaction (Fig. 4J). Further truncation analysis of SLC25A6 showed that deletion of the 211–298 amino acid region (Myc-SLC25A6-ΔD3) abolished its interaction with MRPL13, while truncations in other regions had no effect, confirming that SLC25A6 interacts with MRPL13 through its 211–298aa region (Fig. 4K). These findings provide a detailed molecular understanding of the MRPL13-SLC25 A6 interaction and its potential role in regulating mitochondrial function and OC progression.

### MRPL13 enhances ubiquitin-mediated degradation of SLC25A6

**Fig. 5:**
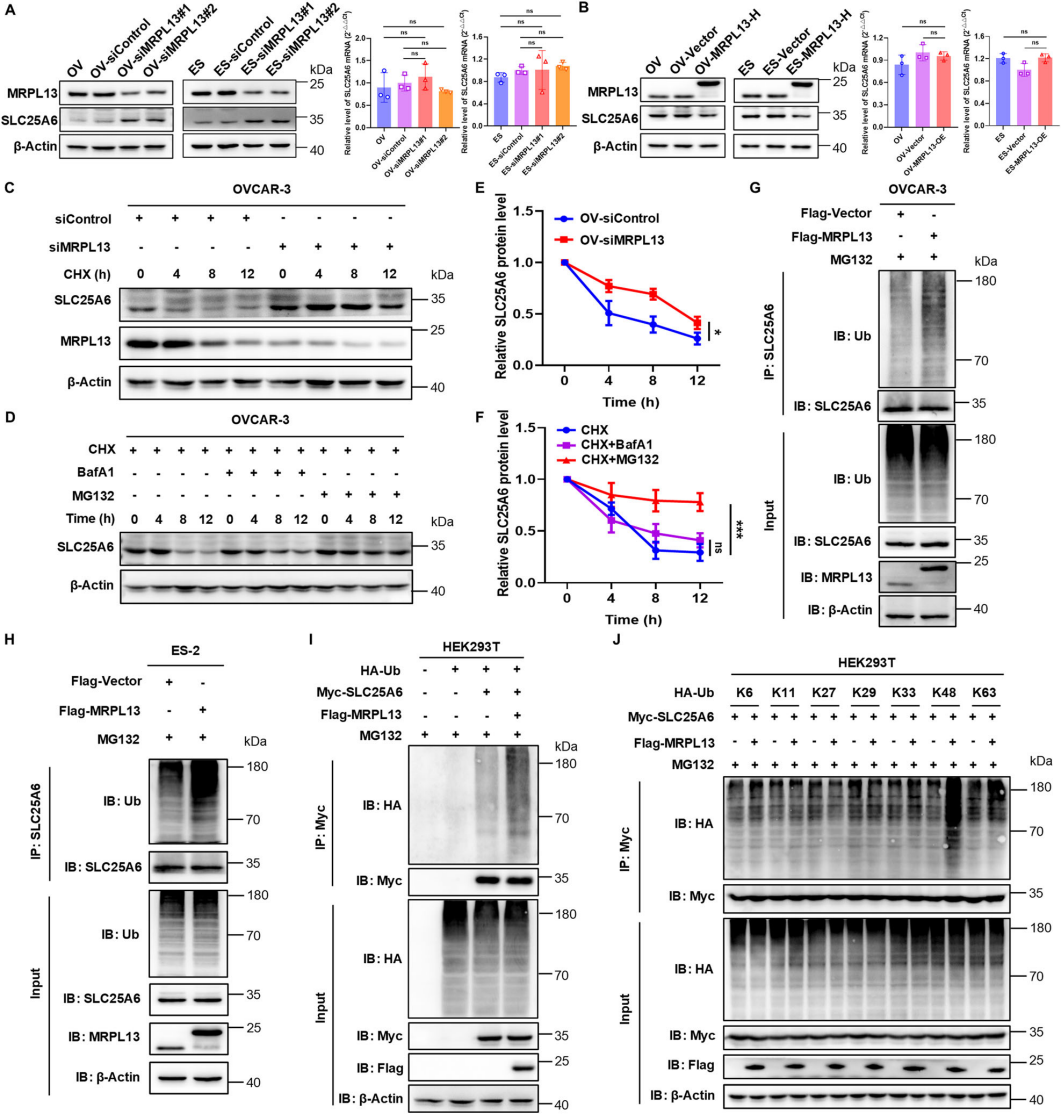
MRPL13 enhances ubiquitin-mediated degradation of SLC25A6. **A**, **B** The protein and mRNA expression levels of SLC25A6 were measured by western blot and qRT-PCR in OVCAR-3 and ES-2 cells with MRPL13 knockdown or overexpression, respectively. β-Actin was used as an internal control. **C** Stability analysis of SLC25A6 protein half-life was validated by western blot in MRPL13-knockdown OVCAR-3 cells after treatment with 20 μg/mL CHX for the indicated times. **D** Stability analysis of SLC25A6 protein half-life was validated by western blot in OVCAR-3 cells after treatment with 20 μg/mL CHX and either 10 μM MG132 or 400 nM BafA1 for the indicated times. **E**, **F** SLC25A6 protein half-life plots were derived by quantifying the relative levels of SLC25A6 to β-actin protein, based on band intensity. **G**, **H** Ubiquitination assay of SLC25A6 in OVCAR-3 and ES-2 cells with MRPL13 overexpression treated with 10 μM MG132 for 6 h. Cell lysates were immunoprecipitated in the presence of anti-SLC25A6 antibodies. Immunoblotting was performed using anti-Ubiquitin and anti-SLC25A6 antibodies. **I** Ubiquitination assay of SLC25A6 in HEK293T cells co-transfected with HA-Ub, Myc-SLC25A6 or Flag-MRPL13 and treated with 10 μM MG-132 for 6 h. Cell lysates were immunoprecipitated in the presence of anti-Myc antibodies. Immunoblotting was performed using anti-HA and anti-Myc antibodies. **J** Ubiquitination assay of SLC25A6 in HEK293T cells co-transfected with Myc-SLC25A6, Flag-MRPL13 or the HA-tagged K6-, K11-, K27-, K29-, K33-, K48-, or K63-only ubiquitin mutant (for example, K6 indicates that all lysines with the exception of K6 were mutated to arginine) and treated with 10 μM MG-132 for 6 h. Cell lysates were immunoprecipitated in the presence of anti-Myc antibodies. Immunoblotting was performed using anti-HA and anti-Myc antibodies. All assays were performed in three independent experiments. Data are presented as mean ± SD. *, *P* < 0.05; **, *P* < 0.01; ***, *P* < 0.001.

To further investigate the interaction between MRPL13 and SLC25A6, we analyzed their regulatory relationship. In both OVCAR-3 and ES-2 cells, MRPL13 knockdown led to increased SLC25A6 protein expression, while MRPL13 overexpression reduced SLC25A6 protein levels (Fig. 5A, B and Supplementary Fig. 4A, B). Interestingly, SLC25 A6 mRNA levels remained unchanged (Fig. 5A, B), suggesting that MRPL13 may regulate SLC25A6 through post-translational modification rather than transcriptional regulation. To assess the impact of MRPL13 on SLC25A6 protein stability, cycloheximide (CHX) pulse-chase assay was performed. Following CHX treatment, SLC25A6 underwent gradual degradation, while MRPL13 knockdown prolonged the half-life of endogenous SLC25A6 protein, indicating that MRPL13 knockdown inhibited SLC25A6 degradation (Fig. 5C, E). Next, we elucidated the degradation pathway of SLC25A6 regulated by MRPL13. Combined treatment with CHX and proteasome inhibitor MG132 effectively blocked SLC25A6 degradation, while CHX combined with autophagy-lysosome inhibitor BafA1 didn’t significantly affect its degradation rate (Fig. 5D, F). These results demonstrate that SLC25A6 degradation primarily depends on the ubiquitin-proteasome pathway. GO and KEGG analyses of MRPL13 co-expressed genes also indicated an association with the ubiquitin-proteasome pathway (Fig. 3A, B). IP-based ubiquitination assays further confirmed the impact of MRPL13 on SLC25A6 ubiquitination. In OVCAR-3 and ES-2 cells, MRPL13 overexpression increased the endogenous ubiquitination level of SLC25A6 (Fig. 5G, H). Exogenous co-expression of Myc-SLC25A6, Flag-MRPL13, and HA-Ub in HEK293T cells showed that Flag-MRPL13 significantly enhanced the ubiquitination level of Myc-SLC25A6 (Fig. 5I). These results indicate that MRPL13 promotes SLC25A6 ubiquitination, mediating its degradation and downregulating its protein level. To identify the linkage type of ubiquitin chains regulated by MRPL13, we performed ubiquitination assays in HEK293T cells using HA-tagged ubiquitin variants specific for K6-, K11-, K27-, K29-, K33-, K48-, and K63-linked chains. MRPL13 overexpression significantly increased K48-linked ubiquitin chains on SLC25A6 compared to other linkage types (Fig. 5J). Collectively, these findings demonstrate that MRPL13 facilitates K48-linked ubiquitination of SLC25A6, mediating its degradation.

### MRPL13 enhances mitochondrial function and promotes OC progression by inhibiting mPTP opening via SLC25A6

**Fig. 6:**
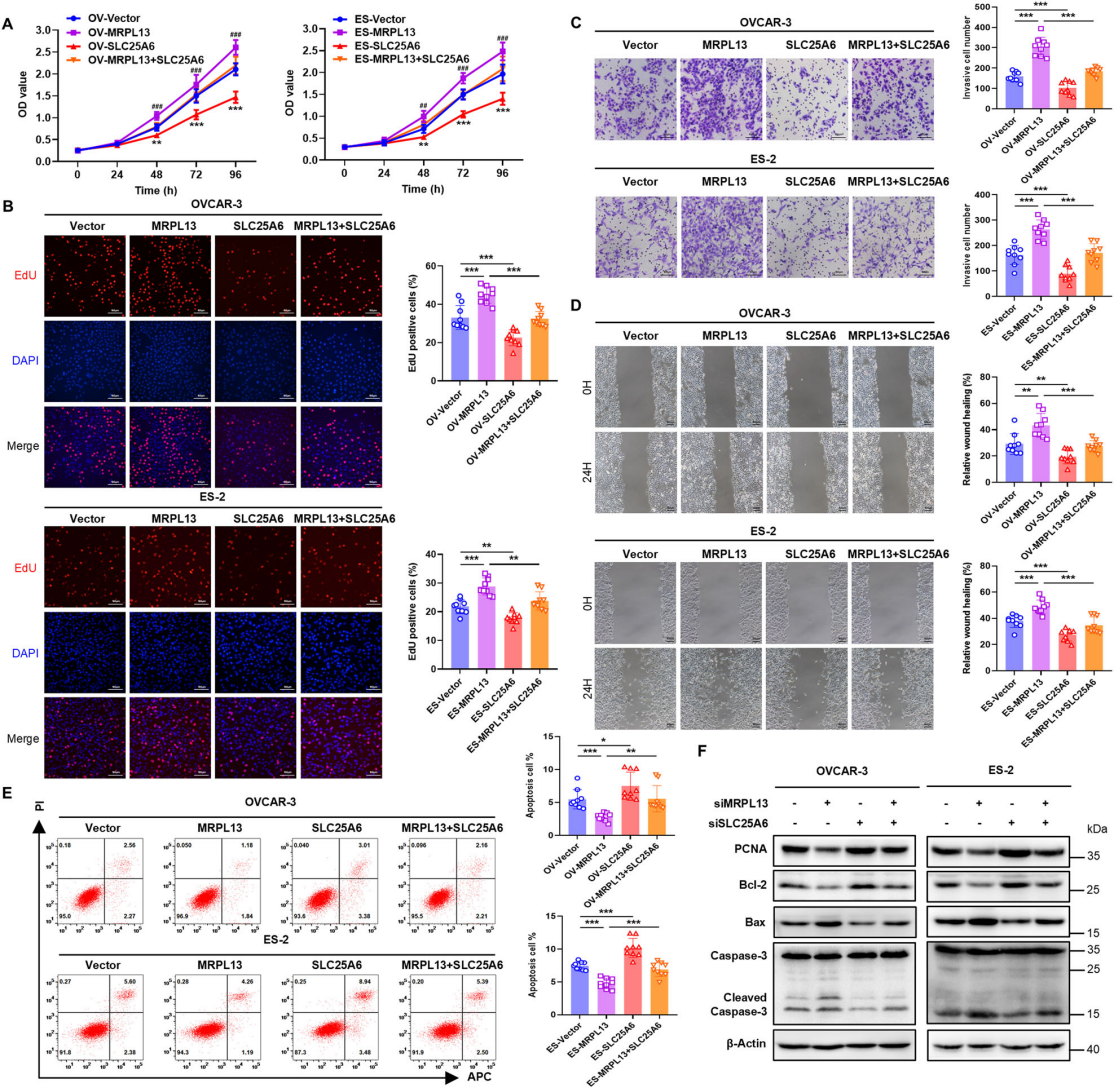
MRPL13 facilitates OC progression by degrading SLC25A6. **A** CCK-8 assays were utilized to evaluate the cell viability in OVCAR-3 and ES-2 cells with MRPL13 and/or SLC25A6 overexpression. *: Vector vs SLC25A6; #: MRPL13 vs MRPL13 + SLC25A6. **B** EdU assays were conducted to assess cell proliferation in OVCAR-3 and ES-2 cells with MRPL13 and/or SLC25A6 overexpression. DAPI (blue) staining was performed to indicate total cells, while EdU (red) incorporation indicated cells with active DNA replication. **C** Transwell assays were conducted to assess cell invasion ability in OVCAR-3 and ES-2 cells with MRPL13 and/or SLC25A6 overexpression. **D** Wound healing assays were used to evaluate cell migration ability in OVCAR-3 and ES-2 cells with MRPL13 and/or SLC25A6 overexpression. **E** Cell apoptosis of OVCAR-3 and ES-2 cells with MRPL13 and/or SLC25A6 overexpression was validated by flow cytometry. **F** Protein expression level of PCNA, Bcl-2, Bax, Caspase-3 and Cleaved Caspase-3 were monitored by western blot for lysates from OVCAR-3 and ES-2 cells with MRPL13 and/or SLC25A6 knockdown. β-Actin was used as an internal control. All assays were performed in three independent experiments. Data are presented as mean ± SD. *, *P* < 0.05; **, *P* < 0.01; ***, *P* < 0.001.

To explore whether MRPL13 promotes the malignant behavior of OC cells through SLC25A6, we performed rescue functional experiments. Ectopic overexpression of SLC25A6 suppressed cell viability, proliferation, invasion, and migration, while increasing apoptosis levels in OC cells (Fig. 6A–E). Moreover, overexpression of SLC25A6 reversed the enhanced cell viability, proliferation, invasion, and migration, as well as the reduced apoptosis, induced by MRPL13 overexpression (Fig. 6A–E). Key proteins regulating proliferation and apoptosis were also examined, showing that knockdown of SLC25A6 partially offset the inhibitory effect on proliferation and the promotion of apoptosis caused by MRPL13 knockdown, which further corroborating the aforementioned conclusions (Fig. 6F and Supplementary Fig. 4C). These results demonstrate that MRPL13 promotes the malignant biological behavior of OC cells through SLC25A6.

**Fig. 7:**
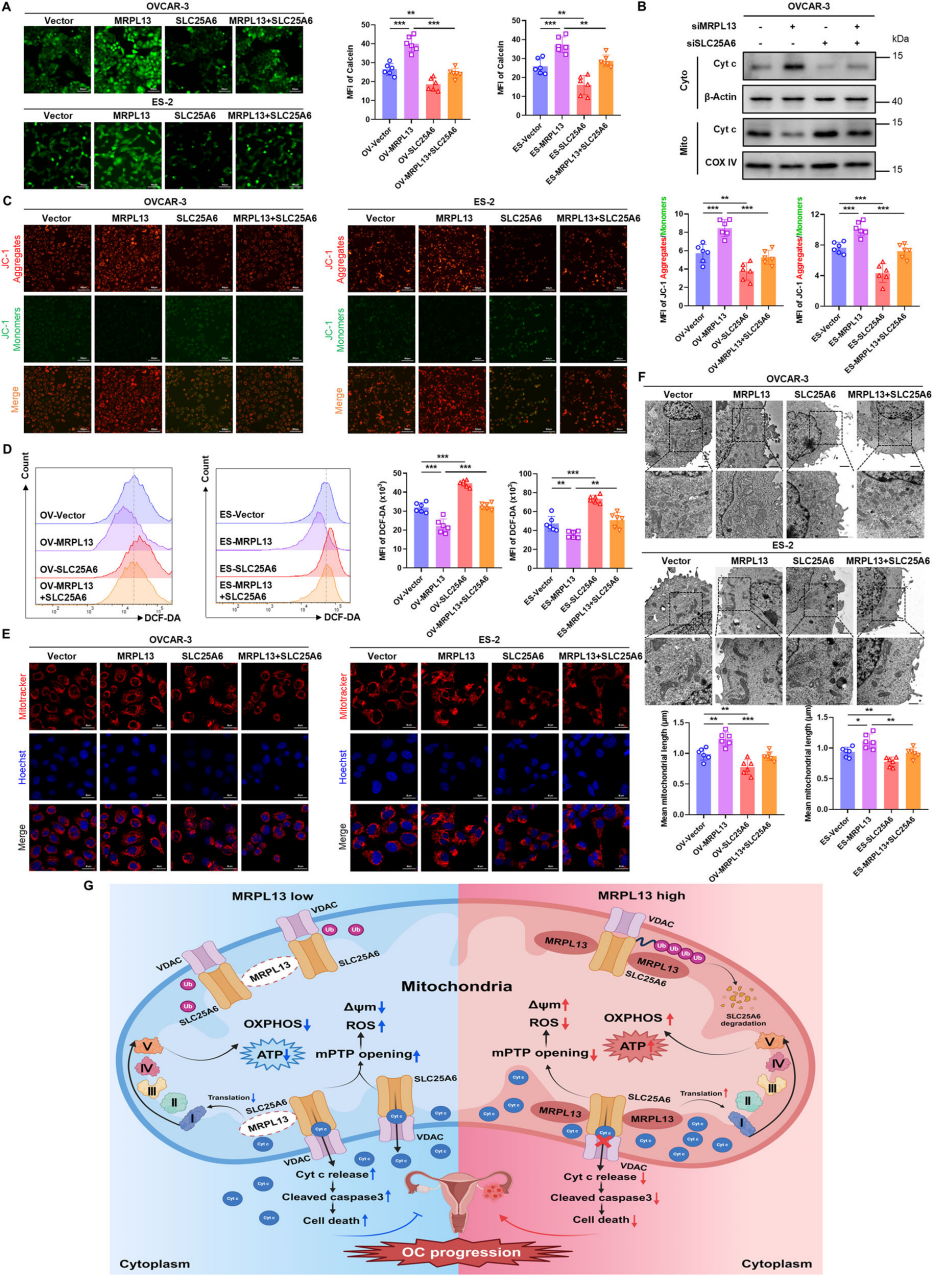
MRPL13 inhibits mPTP opening and improves mitochondrial function through SLC25A6. **A** The degree of mPTP opening in OVCAR-3 and ES-2 cells with MRPL13 and/or SLC25A6 overexpression was detected by quantitative analysis of the MFI of Calcein AM fluorescent probe. **B** Distribution of Cyt c was detected in cytoplasm (Cyto) and mitochondrial (Mito) lysate by western blot. Lysates were collected from OVCAR-3 cells with MRPL13 and/or SLC25A6 knockdown. β-Actin was used as an internal control in cytoplasm. COX IV was used as an internal control in mitochondrial. **C** Mitochondrial membrane potential in OVCAR-3 and ES-2 cells with MRPL13 and/or SLC25A6 overexpression was determined by quantitative analysis of the MFI of JC-1 aggregates (red)/monomers (green). **D** The mean fluorescence intensity (MFI) of DCF-DA was quantitatively calculated by flow cytometry to assess the ROS level in OVCAR-3 and ES-2 cells with MRPL13 and/or SLC25A6 overexpression. **E** Fluorescence staining (Mito-Tracker Red CMXRos, red; Hoechst, blue) showing the mitochondrial morphology in OVCAR-3 and ES-2 cells with MRPL13 and/or SLC25A6 overexpression. The mitochondrial length was quantitatively analyzed by ImageJ Mitochondria Analyzer. **F** Representative images of TEM showing the mitochondrial morphology in OVCAR-3 and ES-2 cells with MRPL13 and/or SLC25A6 overexpression. **G** Schematic depicting the molecular mechanism by which MRPL13 improves mitochondrial function and facilitates OC progression by inhibiting mPTP opening through SLC25A6. All assays were performed in three independent experiments. Data are presented as mean ± SD. *, *P* < 0.05; **, *P* < 0.01; ***, *P* < 0.001.

SLC25A6 plays a profound role in the formation of mPTP, regulating aberrant opening of mPTP and playing a crucial role in mitochondrial function and cell fate. Given our findings that MRPL13 knockdown impacts mitochondrial functions such as mPTP opening in OC cells, we propose to further explore whether MRPL13 inhibits the opening of mPTP through SLC25A6. MPTP opening assays showed that SLC25A6 overexpression promoted aberrant opening of mPTP and reversed the inhibitory effect of MRPL13 overexpression on mPTP opening (Fig. 7A). Sustained mPTP opening leads to Cyt c release from the mitochondrial intermembrane space into the cytoplasm, increased ROS generation, and activation of caspase-dependent apoptotic pathways [32]. Mitochondrial isolation experiments revealed that MRPL13 knockdown increased the release of Cyt c into cytoplasm, while SLC25A6 knockdown ameliorated this effect induced by MRPL13 knockdown (Fig. 7B and Supplementary Fig. 4D). Additionally, SLC25A6 overexpression reversed the MRPL13 overexpression-induced mitochondrial membrane potential elevation and ROS reduction (Fig. 7C, D). MitoTracker staining and TEM analysis showed that SLC25A6 overexpression caused mitochondrial fragmentation, disruption of cristae structure, and decreased mitochondrial mass. SLC25A6 overexpression also reversed the improvement in mitochondrial morphology induced by MRPL13 overexpression (Fig. 7E, F). These findings demonstrate that MRPL13 regulates mitochondrial function in OC cells via SLC25A6.

To further elucidate whether SLC25A6 regulates mitochondrial function and malignant behaviors of OC cells by modulating the opening of mPTP, we performed rescue experiments using the mPTP inhibitor cyclosporin A (CsA). The results demonstrated that CsA partially reversed the activation of abnormal mPTP opening induced by SLC25A6 overexpression, as well as the associated loss of mitochondrial membrane potential and increase in ROS levels (Supplementary Fig. 5A–C). Moreover, CsA treatment partially rescued the inhibitory effect of SLC25A6 overexpression on OC cell proliferation and its pro-apoptotic effect (Supplementary Fig. 5D–F). In summary, these findings indicate that MRPL13 enhances mitochondrial function and promotes OC malignant biological behavior by inhibiting mPTP opening via SLC25A6, highlighting its critical role in OC progression (Fig. 7G).

## Discussion

OC remains the most lethal gynecologic malignancy globally, characterized by its insidious onset, lack of early diagnostic biomarkers, frequent recurrence and metastasis, and resistance to chemotherapy, all of which contribute to its poor prognosis [33, 34]. Thus, there is an urgent need to investigate the molecular mechanisms underlying OC progression, identify potential diagnostic and therapeutic targets, and develop promising clinical interventions. In recent years, accumulating evidence has highlighted the dysregulation of mitochondrial-associated proteins, with mitochondrial dysfunction closely linked to the onset, progression, and metastasis of OC.

MRPs have emerged as critical players in tumor biology. Studies have shown that MRPs are abnormally expressed in various cancers and are associated with poor prognosis and metastatic lesions [16]. With the deepening of research, the role of MRPL13 in tumors has been gradually revealed and understood. MRPL13 is overexpressed in breast cancer, lung cancer, and gastric cancer, serving as a potential predictive biomarker, with its high expression correlated with poor patient prognosis. In breast cancer, MRPL13 promotes cell proliferation, migration, and epithelial-mesenchymal transition via the PI3K/AKT/mTOR pathway, contributing to metastasis and recurrence [35]. In lung adenocarcinoma, single-cell analysis and in vitro experiments have demonstrated that MRPL13 knockdown decreases cell viability, delays tumor proliferation and invasion, and increases apoptosis [36]. However, the role of MRPL13 in OC has not been previously investigated. In this study, we comprehensively elucidated the oncogenic function of MRPL13 in OC for the first time. Across multiple datasets and in the Shengjing Hospital cohort, we found that MRPL13 was highly expressed in OC, and its expression was significantly associated with FIGO staging. High MRPL13 expression was predictive of poor prognosis in OC patients, with MRPL13 expression and FIGO stage identified as independent prognostic factors for survival. Functionally, MRPL13 promoted the proliferation, invasion, and migration of OC cells in vitro, while suppressing apoptosis. In vivo, MRPL13 significantly enhanced ovarian tumor growth. These findings establish the pivotal role of MRPL13 in OC progression and suggest its potential as a prognostic biomarker. Our study sheds light on the importance of MRPL13 in the context of OC development and progression. Given its oncogenic role, MRPL13 may serve not only as a prognostic marker but also as a potential therapeutic target for OC. Further research into the precise molecular pathways regulated by MRPL13 could provide valuable insights for the development of targeted therapies aimed at improving outcomes for OC patients.

As a mitochondrial ribosomal protein, MRPL13 plays a critical role in maintaining the structural and functional integrity of mitochondria [37]. MRPs are essential for the translation of core components of the OXPHOS complexes, making them key regulators of mitochondrial energy metabolism. Additionally, MRPs are involved in regulating cell death pathways and play a central role in maintaining mitochondrial homeostasis. Dysregulation of MRPs compromises mitochondrial function, leading to disrupted energy metabolism and homeostasis, which are hallmarks of malignancy in tumors [38]. While MRPL13 has diverse biological functions that are not yet fully elucidated, our GO and KEGG analyses revealed that MRPL13 expression is closely associated with mitochondrial translation, metabolic pathways, OXPHOS, and apoptotic pathways. GSEA further confirmed a positive correlation between MRPL13 expression and OXPHOS activity. Previous studies have demonstrated that suppressing MRPL13 expression leads to mitochondrial ribosome defects, weakening OXPHOS activity and affecting the invasiveness of hepatocellular carcinoma cells [39]. Similarly, MRPL12 knockdown has been shown to cause mitochondrial structural damage and reduced OXPHOS activity. MRPL12 drives the progression of lung adenocarcinoma by affecting mitochondrial function, specifically mitochondrial OXPHOS [40]. Although the relationship between mitochondrial dysfunction and cancer transformation is increasingly understood, the role of MRPL13 in regulating mitochondrial function and OC progression has remained unclear. Our study sheds light on the critical role of MRPL13 in OC. We found that MRPL13 knockdown significantly impaired mitochondrial function in OC cells, leading to reduced OXPHOS levels, ATP depletion, elevated ROS production, decreased mitochondrial membrane potential, and increased aberrant opening of mPTP. These mitochondrial disruptions ultimately triggered cell death. Additionally, MRPL13 knockdown induced noticeable changes in mitochondrial morphology, including mitochondrial fragmentation, disruption of cristae structure, and decreased mitochondrial mass. These findings suggest that MRPL13 plays a crucial role in regulating mitochondrial energy metabolism and cell death, potentially driving OC malignancy by regulating mitochondrial function.

To further explore the molecular mechanism and potential targets by which MRPL13 promotes OC progression, we identified a novel binding partner of MRPL13, SLC25A6, through mass spectrometry and Co-IP experiments. Molecular docking and immunofluorescence analyses confirmed the specific interaction between MRPL13 and SLC25A6. Notably, we found that MRPL13 influenced the protein stability of SLC25A6 without affecting its mRNA levels. Ubiquitination, a critical post-translational modification, regulates protein degradation and stability [41]. Previous studies have shown that SLC25A6 can promote the ubiquitination-mediated degradation of SLC25A6 by interacting with E3 ubiquitin ligases [42]. Dysregulation of protein ubiquitination and deubiquitination has been closely linked to various diseases, including cancer [43]. K48-linked ubiquitin chains primarily participate in mediating the degradation of substrate proteins by the 26S proteasome and regulate protein stability. Mechanistically, our ubiquitination assays demonstrated that MRPL13 overexpression significantly increased K48-linked ubiquitin chains on SLC25A6, promoting its ubiquitin-proteasome-mediated degradation and reducing SLC25A6 protein levels. These findings have highlighted a novel binding partner for MRPL13, expanding the understanding of ubiquitination-mediated regulation of SLC25A6 protein stability.

SLC25A6 plays a pivotal role in the formation of mPTP, contributing to the maintenance of mitochondrial membrane permeability, regulating cellular energy metabolism, and influencing mitochondrial function. It is also involved in the induction of cell death, thereby impacting tumor progression [23]. Overexpression of SLC25A6 has been shown to induce apoptosis in HeLa cells through the mitochondrial pathway [44]. Similarly, in melanoma cells, SLC25A6 overexpression reduces cell viability, activates the cleavage of c-lamin A and PARP, and triggers cell death [45]. However, the role of SLC25A6 in OC remains unexplored. We have demonstrated that MRPL13 promotes the malignant biological behavior of OC cells by regulating SLC25A6. Given the critical role of SLC25A6 in mPTP formation, further exploration of its downstream complex regulatory network affecting the malignant progression of OC is warranted.

As a critical channel switch regulating cell death, mPTP plays a profound role in the development and progression of various malignancies [46, 47]. Our results indicate that MRPL13 can affect mitochondrial functions such as abnormal opening of mPTP, Cyt c release, mitochondrial membrane potential, and ROS levels by regulating SLC25A6. Persistent aberrant mPTP opening increases mitochondrial permeability, leading to loss of mitochondrial membrane potential, mitochondrial swelling, rupture of the outer membrane and massive release of ROS [25]. Furthermore, aberrant mPTP opening results in the excessive liberation of pro-apoptotic factors, such as Cyt c, which initiate caspase cascade reactions and ultimately lead to cell death [48, 49]. Therefore, based on this evidence, we hypothesize that the MRPL13-SLC25A6 axis may influence mitochondrial function and the malignant progression of OC by regulating the opening of mPTP. However, the interpretation of research findings needs to be considered in the context of key controversies in the field. Pavlov’s group has demonstrated that cells lacking ANT or ATP synthase exhibit CsA-sensitive mitochondrial depolarization without forming the high-conductance mPTP [50, 51]. This not only highlights the critical role of ANT in mPTP regulation but also suggests the existence of mPTP-independent mechanisms for mitochondrial membrane potential depletion. Furthermore, the release of Cyt c can occur through multiple pathways, including mPTP opening and mitochondrial outer membrane permeabilization (MOMP) [52]. Therefore, we need to further strengthen the evaluation of evidence for mPTP involvement in the MRPL13-SLC25A6 axis, promoting the malignant progression of OC.

Notably, our rescue experiments demonstrated that mPTP inhibitor CsA can partially reverse the effects caused by SLC25A6 overexpression, including increased abnormal opening of mPTP, decreased mitochondrial membrane potential, elevated ROS levels, suppressed proliferative capacity, and enhanced apoptotic activation. Targeting mPTP has been proposed as a promising therapeutic strategy for tumor treatment. Similar to our findings, studies have shown that Mortalin can regulate mitochondrial membrane permeability and membrane potential by inhibiting the interaction between SLC25A6 and CypD, which suppresses mPTP opening, ultimately promoting the survival of BRAF-mutant tumor cells [46]. Similarly, studies have demonstrated that EFHD1 inhibits mPTP opening and enhances mitochondrial function through SLC25A6, reducing mitochondrial Cyt c release and promoting the proliferation and chemoresistance in osteosarcoma [47]. In this study, we provide evidence that MRPL13 enhances mitochondrial function and promotes tumor progression in OC by inhibiting mPTP opening via SLC25A6. This study offers new insights into the molecular mechanisms involved in tumor progression mediated by mPTP and lays the foundation for the development of precision therapy strategies based on the MRPL13-SLC25A6-mPTP axis. Future investigations should employ integrated multidimensional strategies that combine conditional knockout models of mPTP-related regulatory factors (e.g., F_1_F_O_ ATP synthase, CypD, VDAC, PiC, and TSPO) with advanced dynamic imaging techniques for mitochondrial ultrastructure analysis, thereby systematically elucidating the spatiotemporal regulatory mechanisms of mPTP in tumorigenesis.

However, our study has certain limitations. First, although we identified the interaction domains of MRPL13 and SLC25A6 through Co-IP experiments with protein truncation constructs, we did not screen small-molecule compounds or design peptides targeting these interaction domains as potential therapeutic agents. Second, while we demonstrated that MRPL13 enhances ubiquitin-mediated degradation of SLC25A6, the specific E3 ubiquitin ligase interacting with SLC25A6 and its precise molecular mechanism remain to be elucidated.

In conclusion, our study identifies a novel oncogenic factor, MRPL13, in OC. MRPL13 is highly expressed in OC tissues and associated with poor prognosis. MRPL13 acts as a potential target for regulating mitochondrial function and OC malignant progression. Mechanistically, MRPL13 interacts with SLC25A6, facilitating the K48-linked ubiquitination of SLC25A6 and mediating its degradation. Our findings elucidate the pathway in which MRPL13 inhibits mPTP opening via SLC25A6, thereby enhancing mitochondrial function and promoting OC progression. Overall, by targeting the MRPL13-SLC25A6-mPTP axis, our study provides valuable insights into the molecular mechanisms underlying OC progression and lays the foundation for development of precise and innovative strategies for targeted therapy.

## Supplementary information

Supplementary Materials

Original Images for BlotsGels

## Data Availability

The data that support the findings of this study are provided within the manuscript or supplementary information files. All relevant data supporting this study’s findings are available from the corresponding author upon request.
