# Supplementary material for: MRPL13 enhances mitochondrial function and promotes tumor progression in ovarian cancer by inhibiting mPTP opening via SLC25A6
Source: Cell Death Dis. 2025 Aug 21;16(1):634. doi: 10.1038/s41419-025-07953-x (PMC12371087; doi:10.1038/s41419-025-07953-x)
Supplement: Supplementary file 1 — Supplementary Materials [file 41419_2025_7953_MOESM1_ESM.docx]

**Supplementary Information**

**MRPL13 enhances mitochondrial function and promotes tumor progression in ovarian cancer by inhibiting mPTP opening via SLC25A6**

Ouxuan Liu^1,2^, Yuexin Hu^1,2^, Shuang Wang^3^, Xin Nie^1,2^, Yuxuan Wang^1,2^, Xiangcheng Fan^1,2^, Kai Zeng^1,2^, Xiao Li^1,2^, Bingying Liu^1,2^, Bei Lin^1,2*^

1. Department of Obstetrics and Gynecology, Shengjing Hospital of China Medical University, Shenyang, Liaoning, 110004, China.

2. Key Laboratory of Obstetrics and Gynecology of Higher Education of Liaoning Province, Key Laboratory of Gynecologic Oncology of Liaoning Province, Shenyang, Liaoning, 110004, China.

3. Department of Obstetrics and Gynecology, Tianjin Central Hospital of Gynecology Obstetrics, Tianjin, 300000, China.

* Corresponding author: Bei Lin, Department of Obstetrics and Gynecology, Shengjing Hospital Affiliated to China Medical University, No. 36, Sanhao Street, Heping District, Shenyang, Liaoning, 110004, China, Email: linbei88@hotmail.com; blin@cmu.edu.cn

**Supplementary Figures**


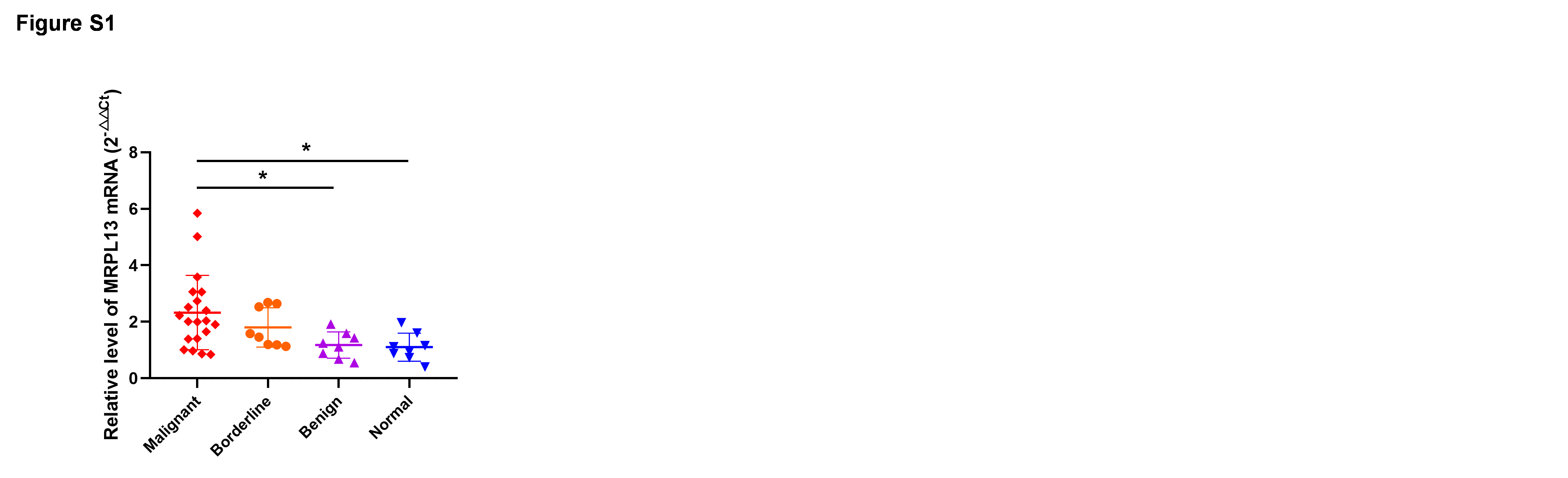


**Supplementary Fig. 1 MRPL13 mRNA expression in ovarian tissues.**

qRT-PCR analysis of MRPL13 mRNA expression in ovarian malignant tumors (n=20), ovarian borderline tumors (n=8), ovarian benign tumors (n=8) and ovarian normal tissues (n=8). β-Actin was used as an internal control. *, *P* < 0.05.


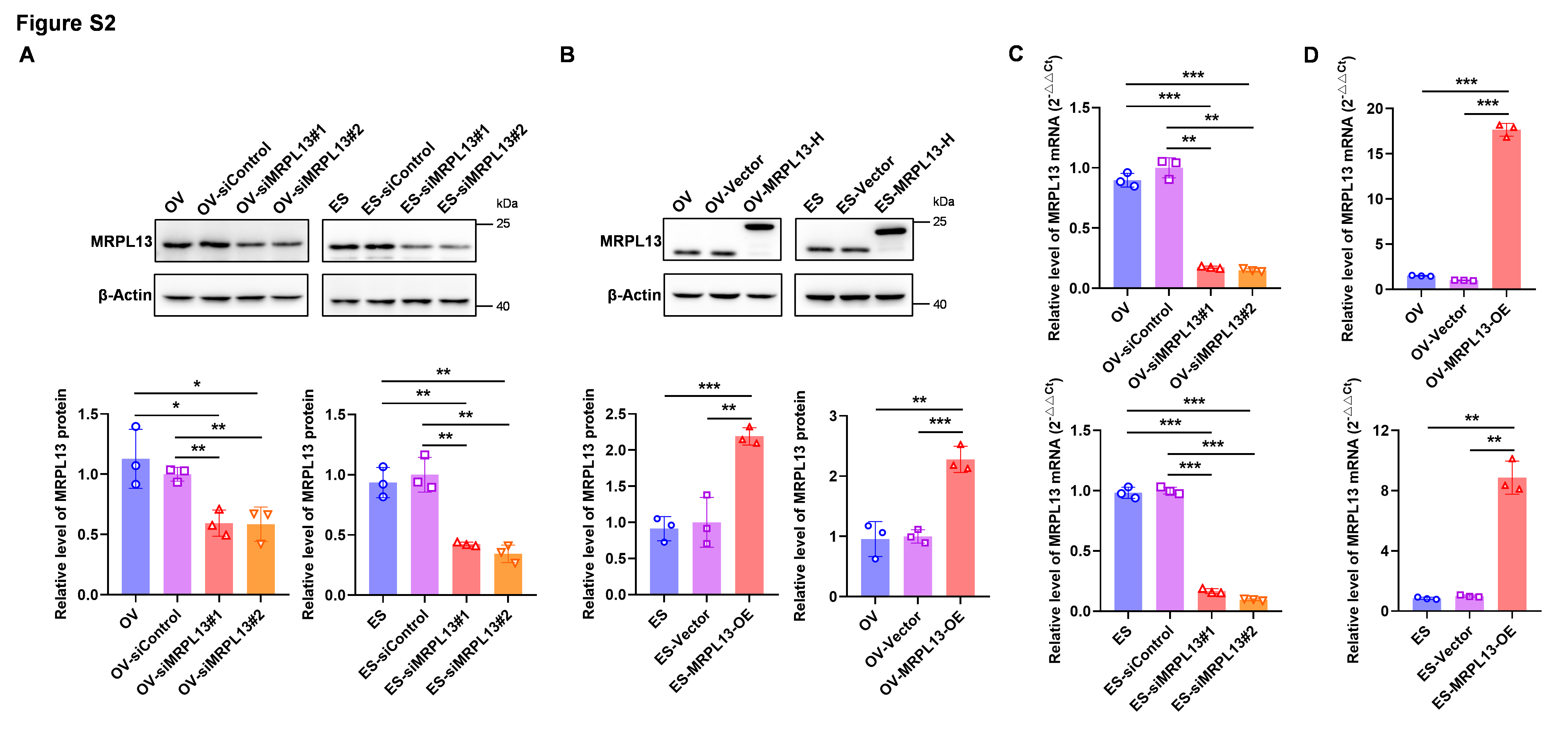


**Supplementary Fig. 2 Validation of MRPL13 knockdown and overexpression efficiency in OC cells.**

A, B Western blot analysis of MRPL13 protein expression in OVCAR-3 and ES-2 cells following knockdown or overexpression. C, D qRT-PCR analysis of MRPL13 mRNA expression in OVCAR-3 and ES-2 cells following knockdown or overexpression. β-Actin was used as an internal control. All assays were performed in three independent experiments. Data are presented as mean±SD. *, *P* < 0.05; **, *P* < 0.01; ***, *P* < 0.001.

**
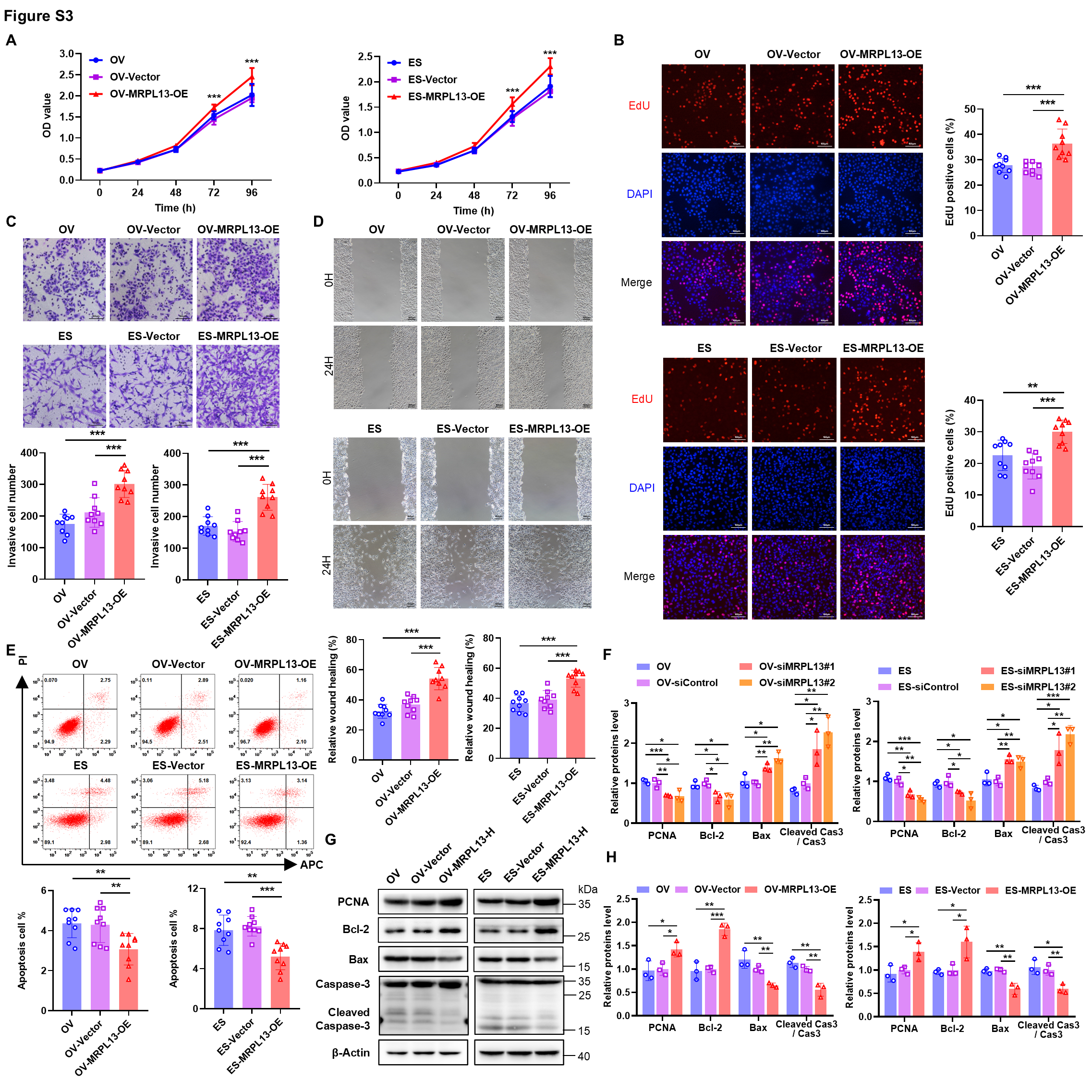
**

**Supplementary Fig. 3 MRPL13 promotes malignant progression of OC cells.**

A CCK-8 assays were utilized to evaluate the cell viability in OVCAR-3 and ES-2 cells with MRPL13 overexpression. *: Vector vs MRPL13-OE. B EdU assays were conducted to assess cell proliferation in OVCAR-3 and ES-2 cells with MRPL13 overexpression. DAPI (blue) staining was performed to indicate total cells, while EdU (red) incorporation indicated cells with active DNA replication. C Transwell assays were conducted to assess cell invasion ability in OVCAR-3 and ES-2 cells with MRPL13 overexpression. D Wound healing assays were used to evaluate cell migration ability in OVCAR-3 and ES-2 cells with MRPL13 overexpression. E Cell apoptosis of OVCAR-3 and ES-2 cells in the MRPL13 overexpression groups were validated by flow cytometry. F Quantitative analysis of western blot results shown in Fig. 2F. G Protein expression level of PCNA, Bcl-2, Bax, Caspase-3 and Cleaved Caspase-3 were monitored by western blot for lysates from OVCAR-3 and ES-2 cells with MRPL13 overexpression. β-Actin was used as an internal control. H Quantitative analysis of western blot results shown in Supplementary Fig. 3G. All assays were performed in three independent experiments. Data are presented as mean±SD. *, *P* < 0.05; **, *P* < 0.01; ***, *P* < 0.001.

**
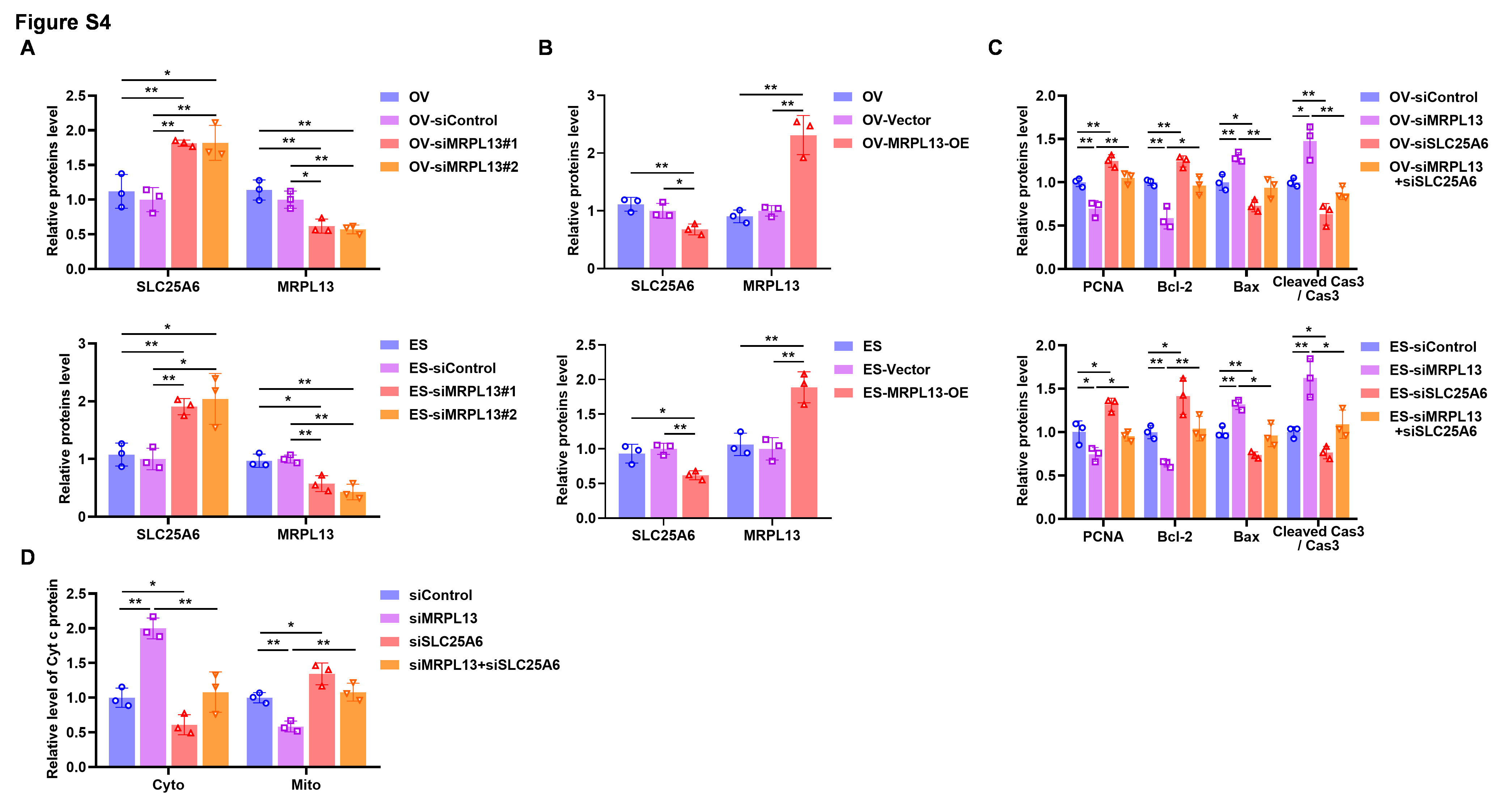
**

**Supplementary Fig. 4 Quantitative analysis of western blot results.**

A Quantitative analysis of western blot results shown in Fig. 5A. B Quantitative analysis of western blot results shown in Fig. 5B. C Quantitative analysis of western blot results shown in Fig. 6F. D Quantitative analysis of western blot results shown in Fig. 7B. Data are presented as mean±SD. *, *P* < 0.05; **, *P* < 0.01.


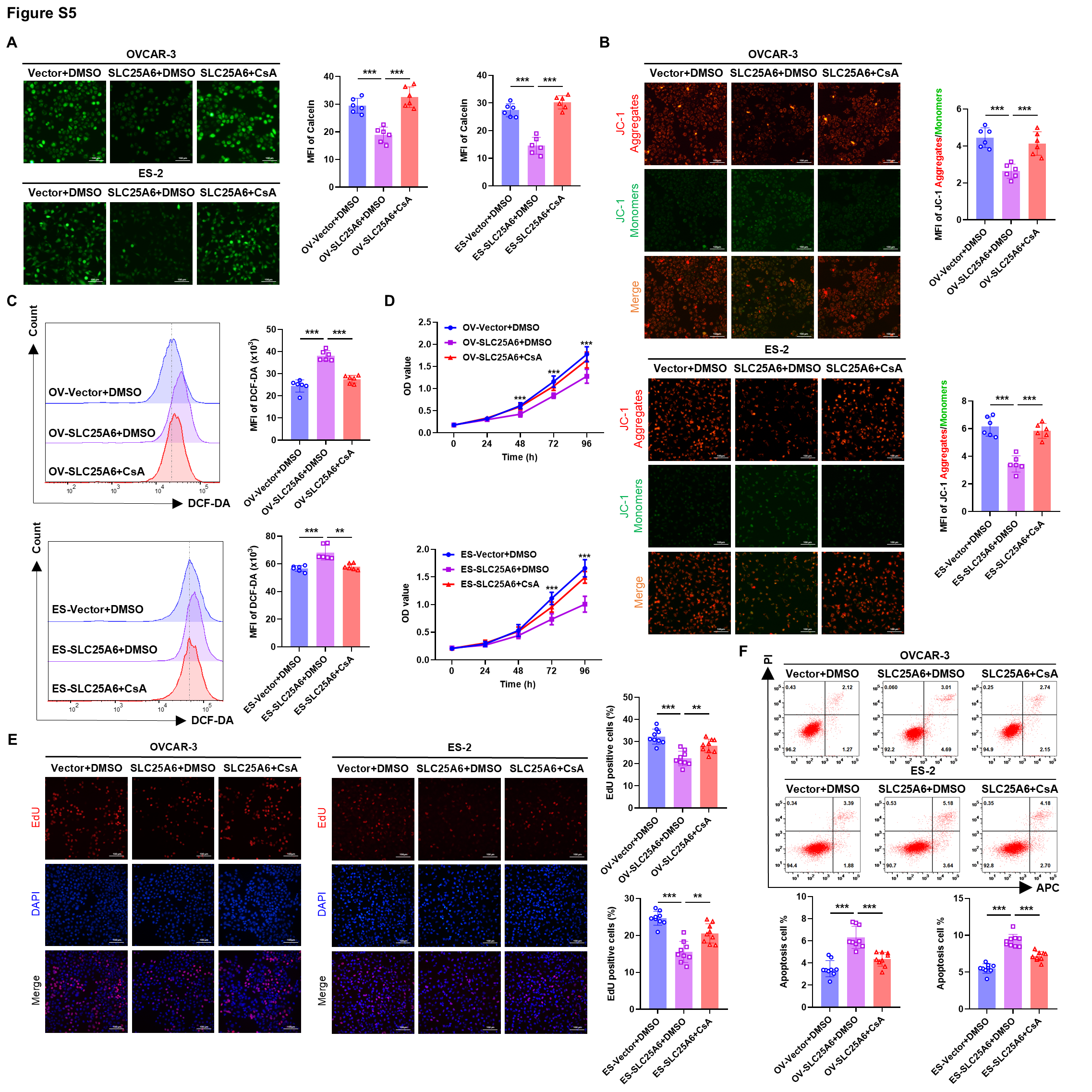


**Supplementary Fig. 5 SLC25A6 regulates mitochondrial function and** **malignant progression in OC cells by modulating mPTP opening.**

A The degree of mPTP opening was assessed by quantitative analysis of the MFI of Calcein AM in SLC25A6-overexpressing OVCAR-3 and ES-2 cells treated with or without CsA. B Mitochondrial membrane potential was determined by quantitative analysis of the MFI of JC-1 aggregates (red) / monomers (green) in SLC25A6-overexpressing OVCAR-3 and ES-2 cells treated with or without CsA. C ROS levels were quantified by flow cytometric analysis of the MFI of DCF-DA in SLC25A6-overexpressing OVCAR-3 and ES-2 cells treated with or without CsA. D CCK-8 assays were utilized to evaluate the cell viability in SLC25A6-overexpressing OVCAR-3 and ES-2 cells treated with or without CsA. *: SLC25A6+DMSO vs SLC25A6+CsA. E EdU assays were conducted to assess cell proliferation in SLC25A6-overexpressing OVCAR-3 and ES-2 cells treated with or without CsA. DAPI (blue) staining was performed to indicate total cells, while EdU (red) incorporation indicated cells with active DNA replication. F Cell apoptosis was validated by flow cytometry in SLC25A6-overexpressing OVCAR-3 and ES-2 cells treated with or without CsA. All assays were performed in three independent experiments. Data are presented as mean±SD. **, *P* < 0.01; ***, *P* < 0.001.

**Supplementary Table 1. The primers and sequences used in the study.**

| Target | Type | Sequence (5’-3’) |
| --- | --- | --- |
| Negative Control | siControl | UUCUCCGAACGUGUCACGUTT |
|  |  | ACGUGACACGUUCGGAGAATT |
| MRPL13 | siRNA#1 | GAAUUUAGUAGAGGAGCUUTT |
|  |  | AAGCUCCUCUACUAAAUUCTT |
| MRPL13 | siRNA#2 | GGACUCCACCUGAAGAUUATT |
|  |  | UAAUCUUCAGGUGGAGUCCTT |
| SLC25A6 | siRNA | GCAACCUUGCCAACGUCAUTT |
|  |  | AUGACGUUGGCAAGGUUGCTT |
| MRPL13 | qPCR primers | F: CTGTGTACCATGCACTGAGTGACTG |
|  |  | R: CTTGTCTAAATCCACCTGGGTAGCC |
| SLC25A6 | qPCR primers | F: AGCCCTCAACTTCGCCTTCAAG |
|  |  | R: AAGTACCTCCAGAACTGCGTGTG |
| ACTB | qPCR primers | F: CAGCAAGCAGGAGTATGACG |
|  |  | R: TTAGGATGGCAAGGGACTTC |
