# Supplementary material for: MRPL13 enhances mitochondrial function and promotes tumor progression in ovarian cancer by inhibiting mPTP opening via SLC25A6
Source: Cell Death Dis. 2025 Aug 21;16(1):634. doi: 10.1038/s41419-025-07953-x (PMC12371087; doi:10.1038/s41419-025-07953-x)

Fig. 2F

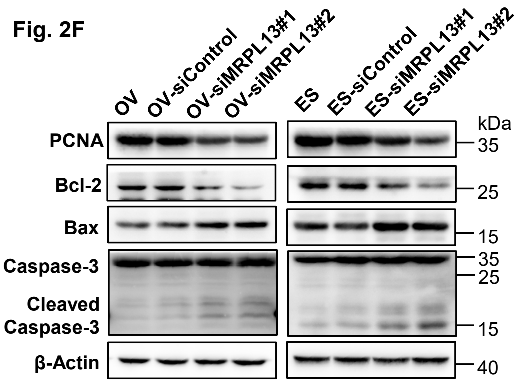

PCNA

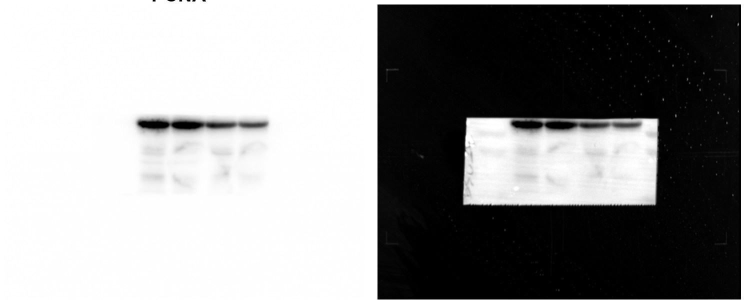

PCNA

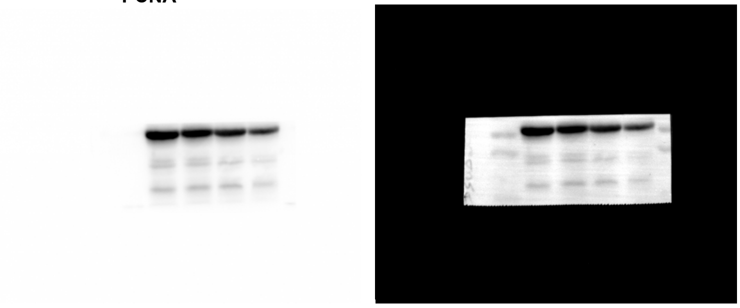

Bcl-2

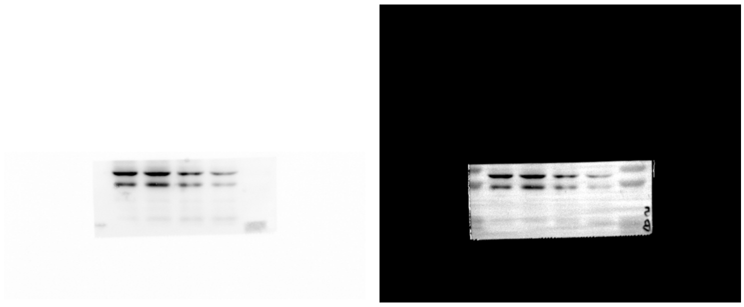

Bcl-2

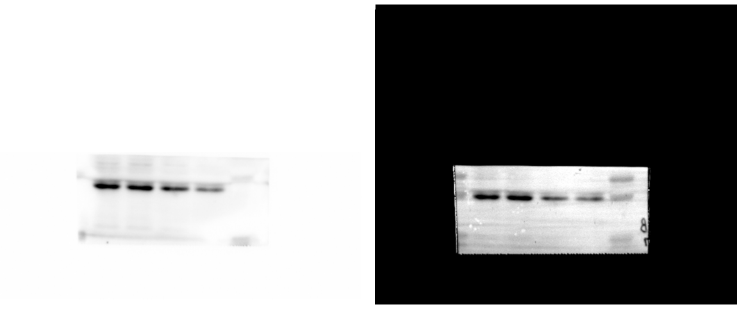

Bax

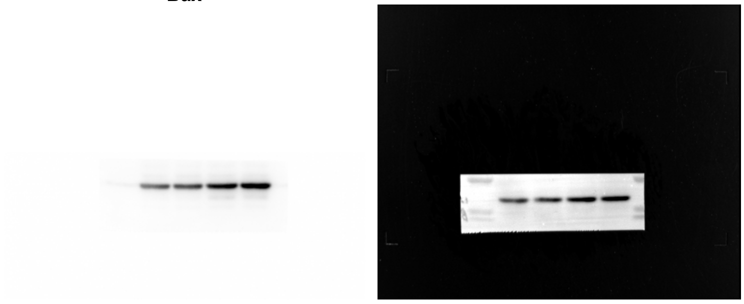

Bax

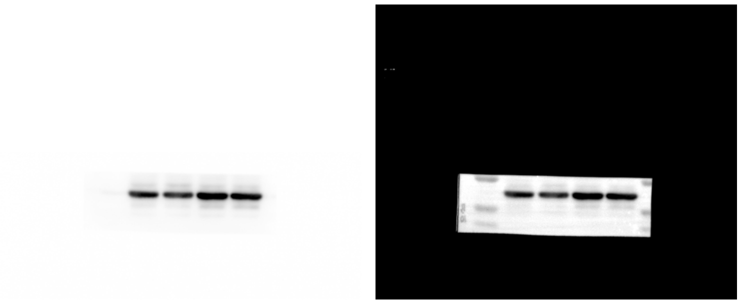

Caspase-3, Cleaved Caspase-3

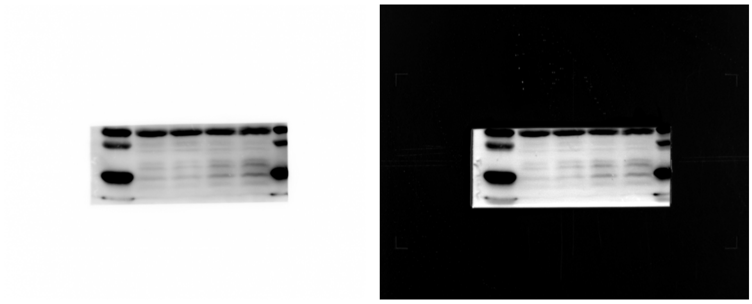

Caspase-3, Cleaved Caspase-3

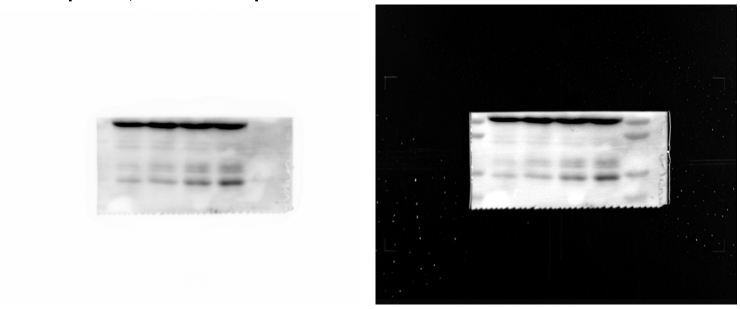

β-Actin

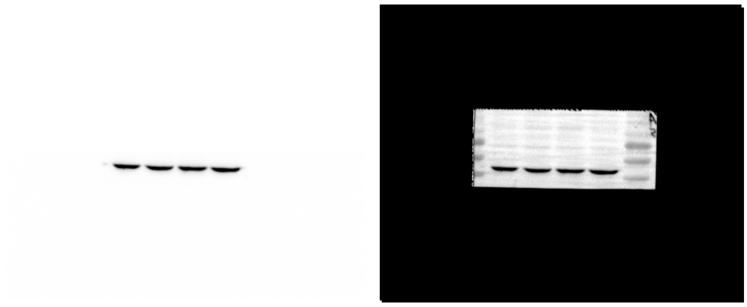

β-Actin

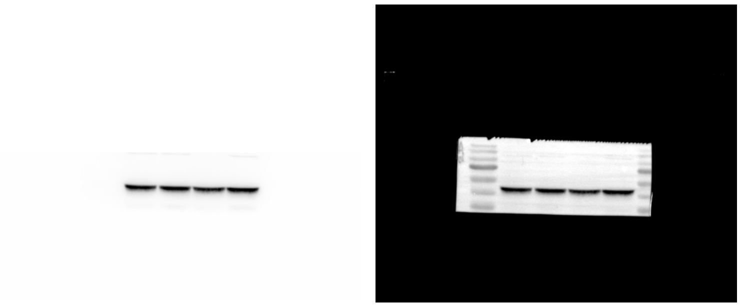

Fig. 4D

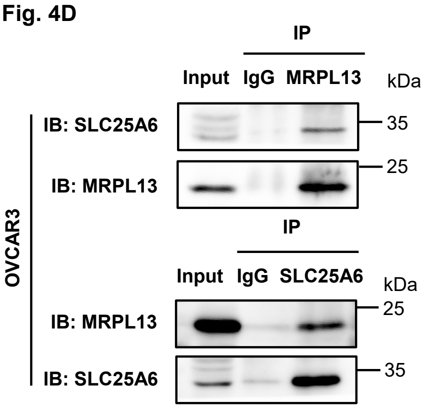

IB: SLC25A6

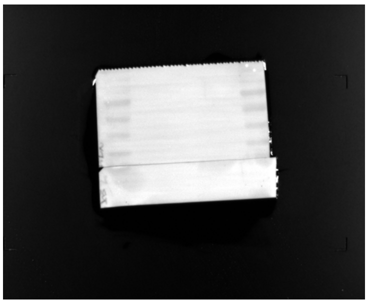
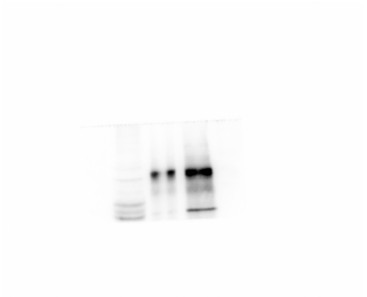
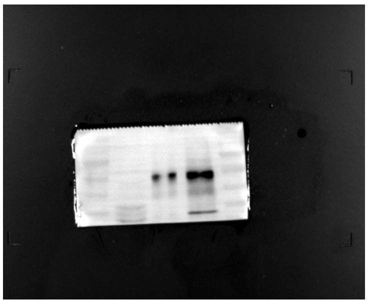

IB: MRPL13

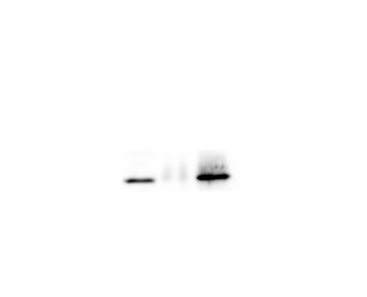
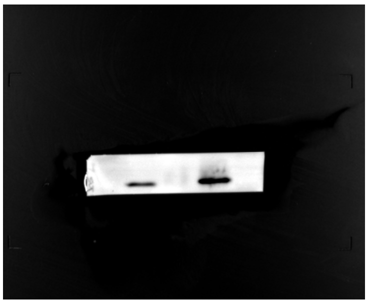

IB: SLC25A6

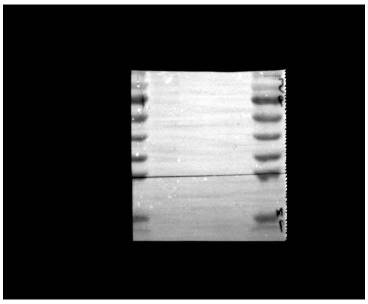
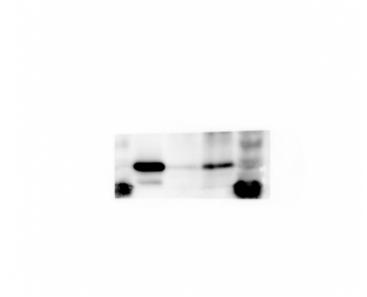
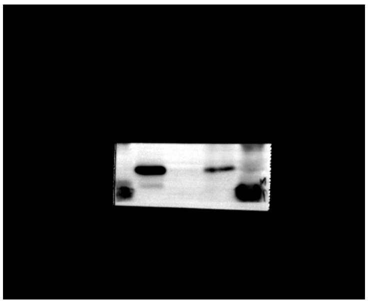

IB: MRPL13

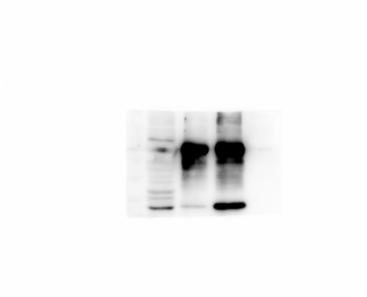
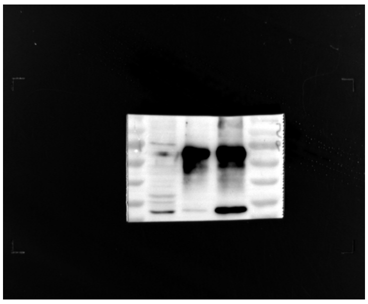

Fig. 4E

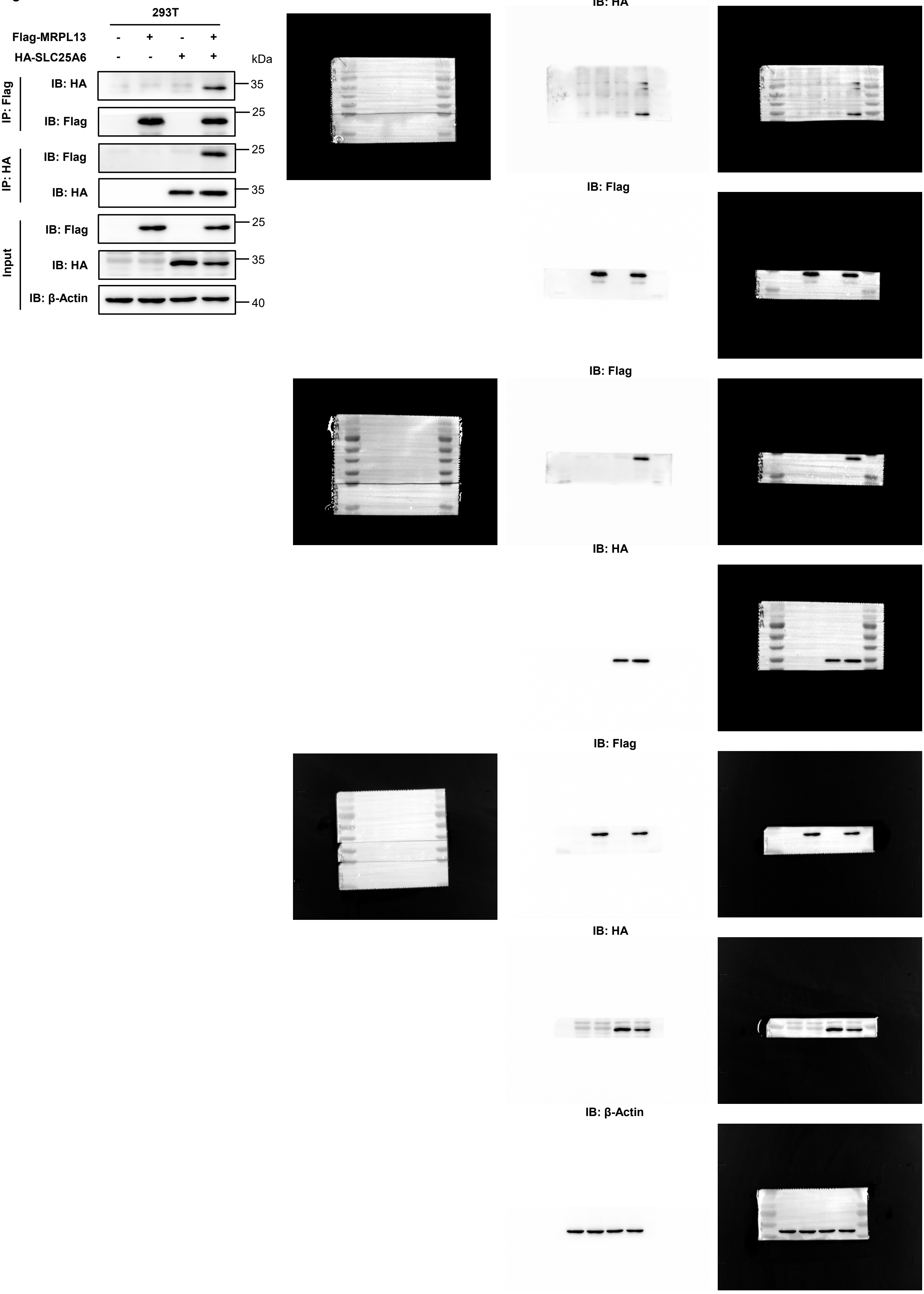
293T
Flag-MRPL13 - + - +
HA-SLC25A6 - - + +
kDa
IP: Flag
IB: HA
35
IB: Flag
25
IP: HA
IB: Flag
25
IB: HA
35
Input
IB: Flag
25
IB: HA
35
IB: β-Actin
40
IB: HA
IB: Flag
IB: Flag
IB: HA
IB: Flag
IB: HA
IB: β-Actin

Fig. 4J

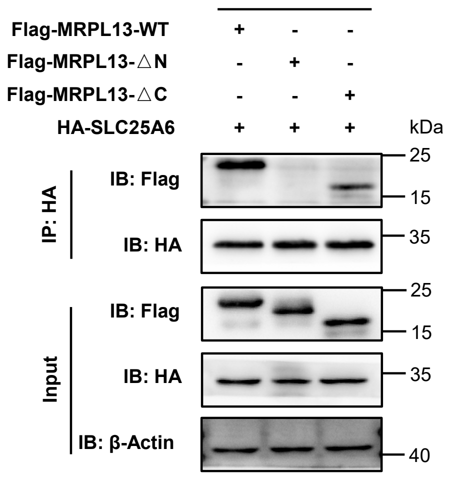

IB: Flag

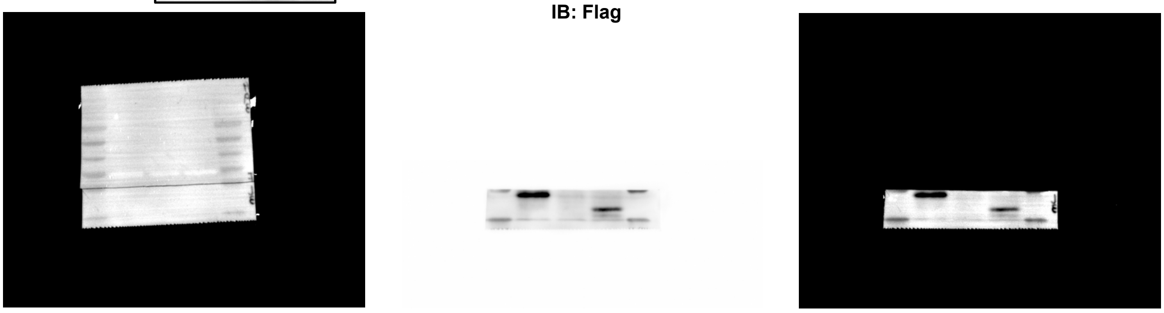

IB: HA

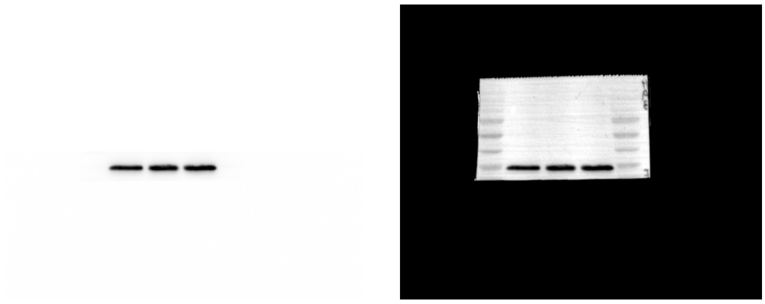

IB: Flag

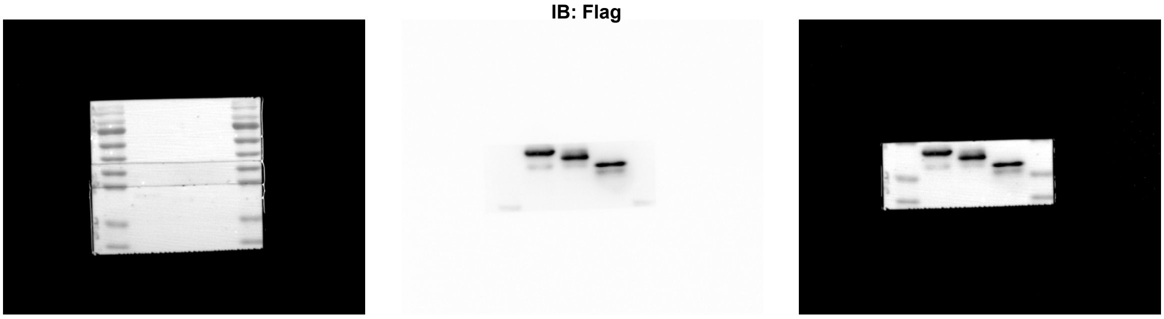

IB: HA

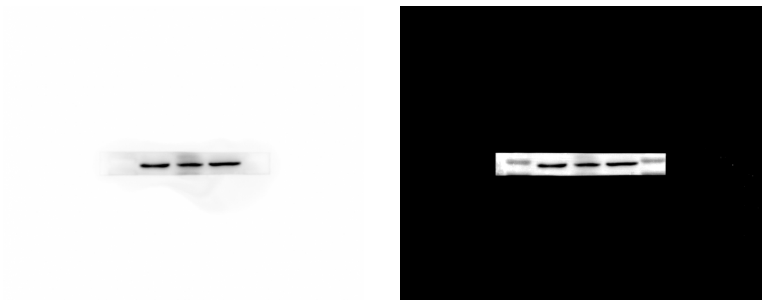

IB: β-Actin

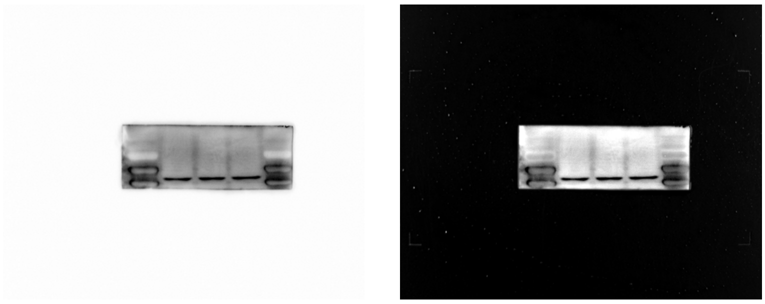

Fig. 4K

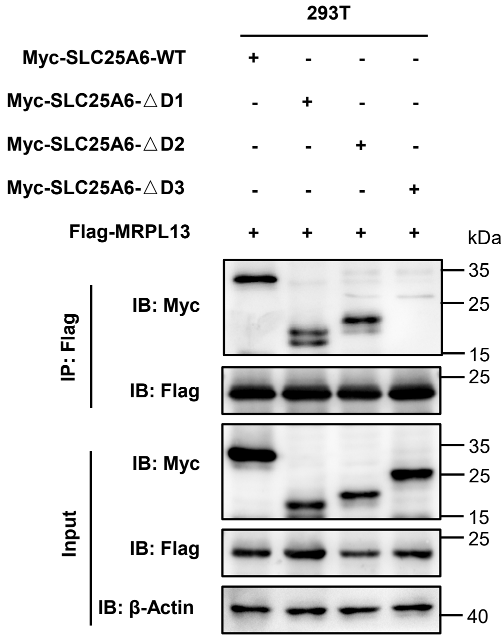

IB: Myc

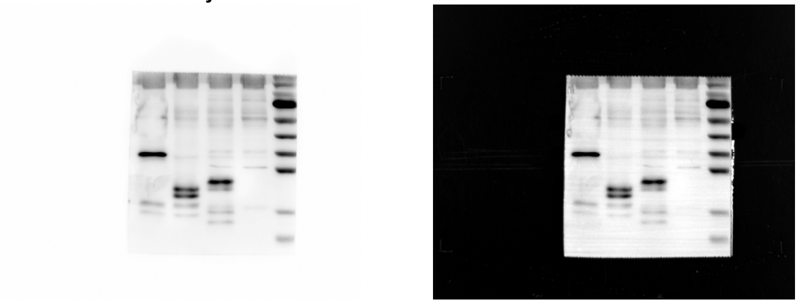

IB: Flag

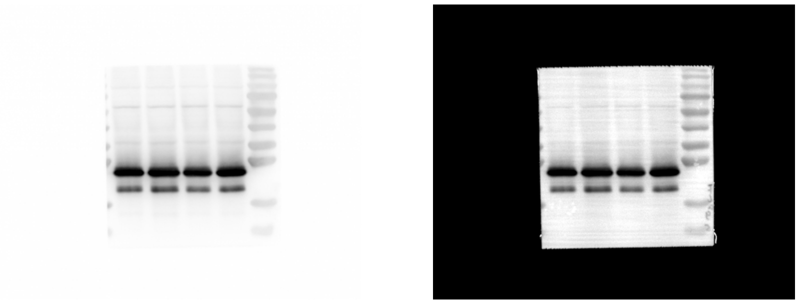

IB: Myc

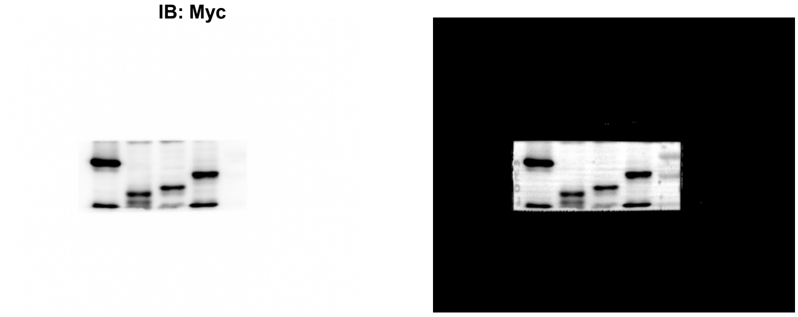

IB: Flag

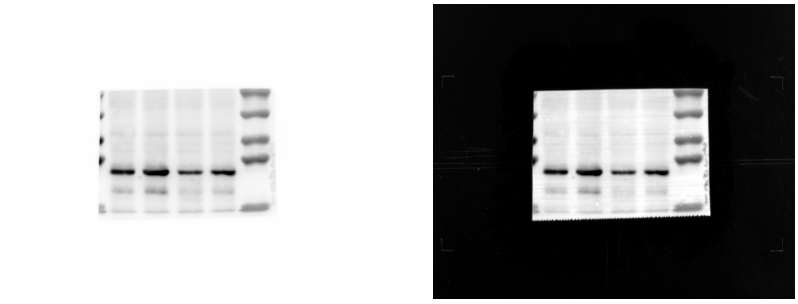

IB: β-Actin

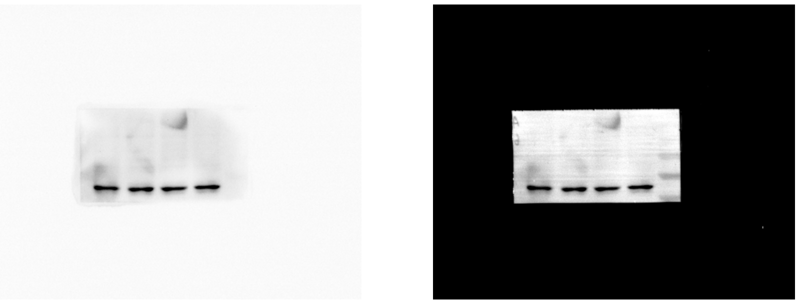

Fig. 5A

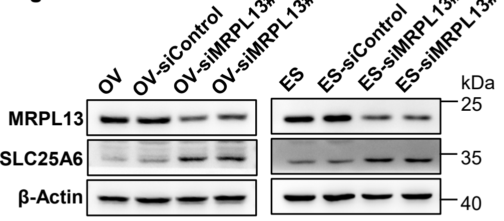

MRPL13

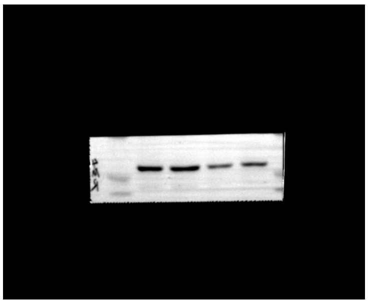

MRPL13

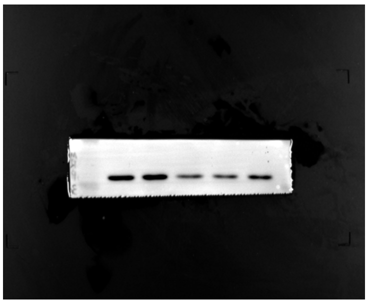

SLC25A6

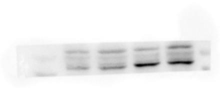

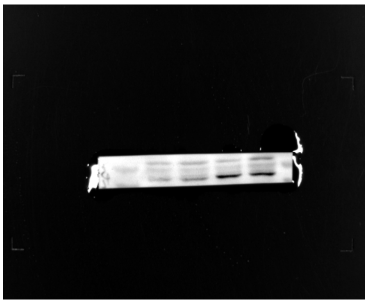

SLC25A6

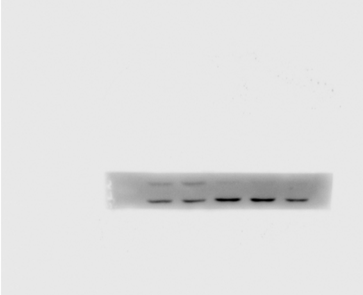

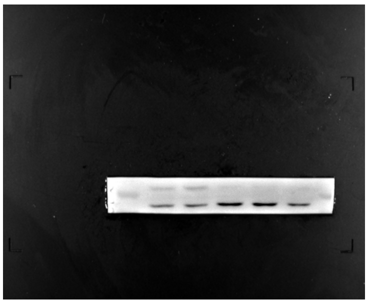

β-Actin

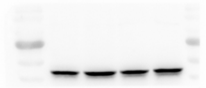

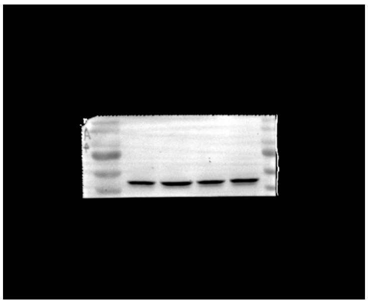

β-Actin

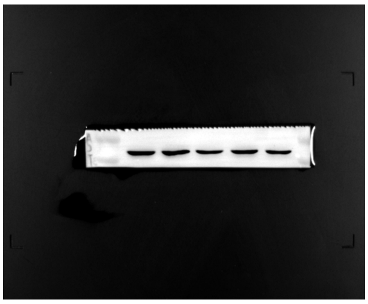

Fig. 5B

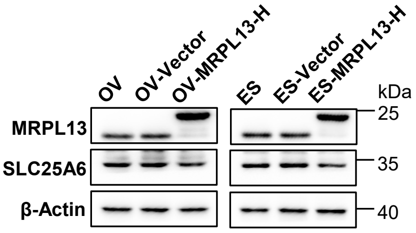
OV
OV-Vector
OV-MRPL13-H
ES
ES-Vector
ES-MRPL13-H
kDa
MRPL13
SLC25A6
β-Actin
25
35
40

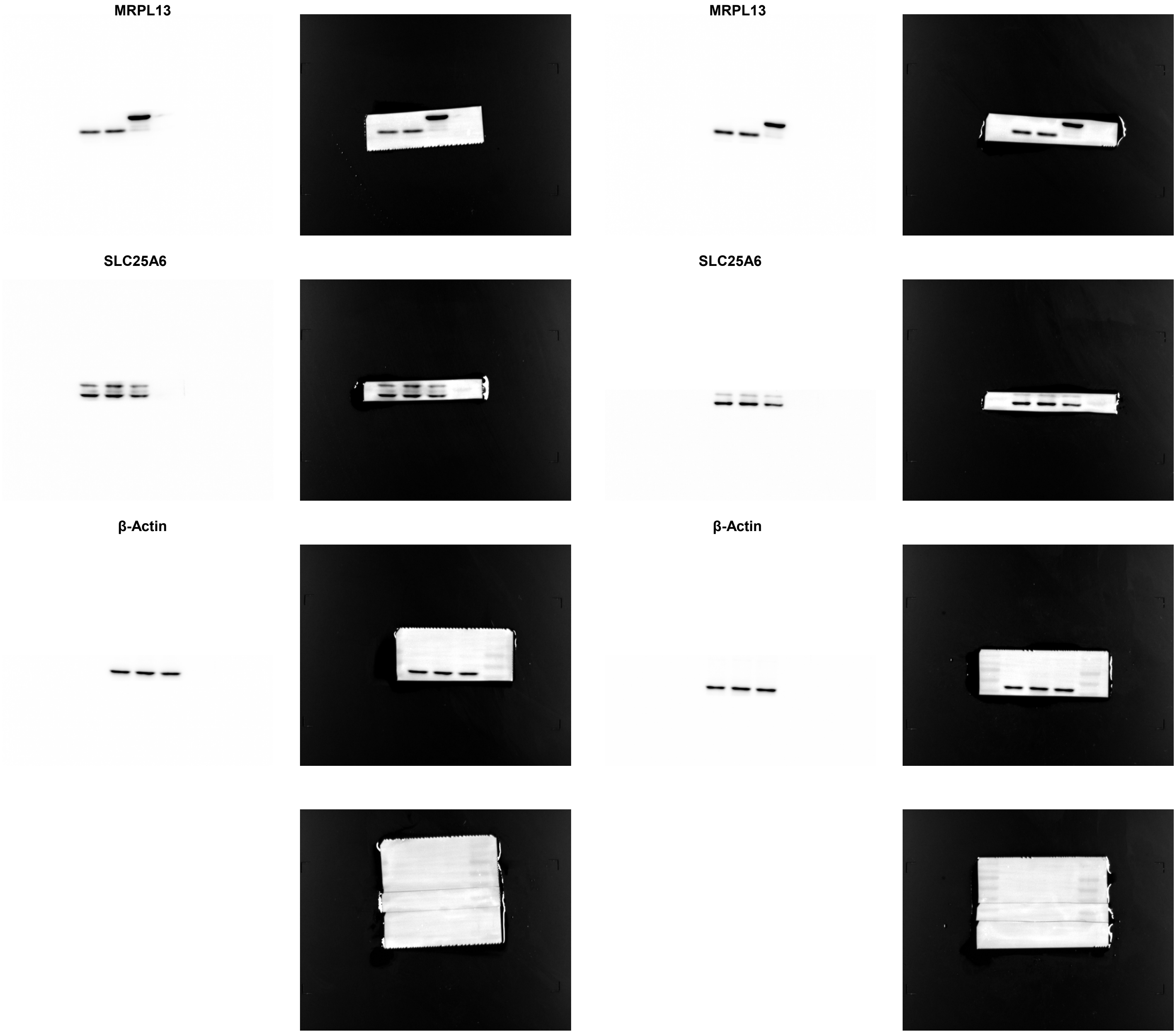
MRPL13
MRPL13
SLC25A6
SLC25A6
β-Actin
β-Actin

Fig. 5C

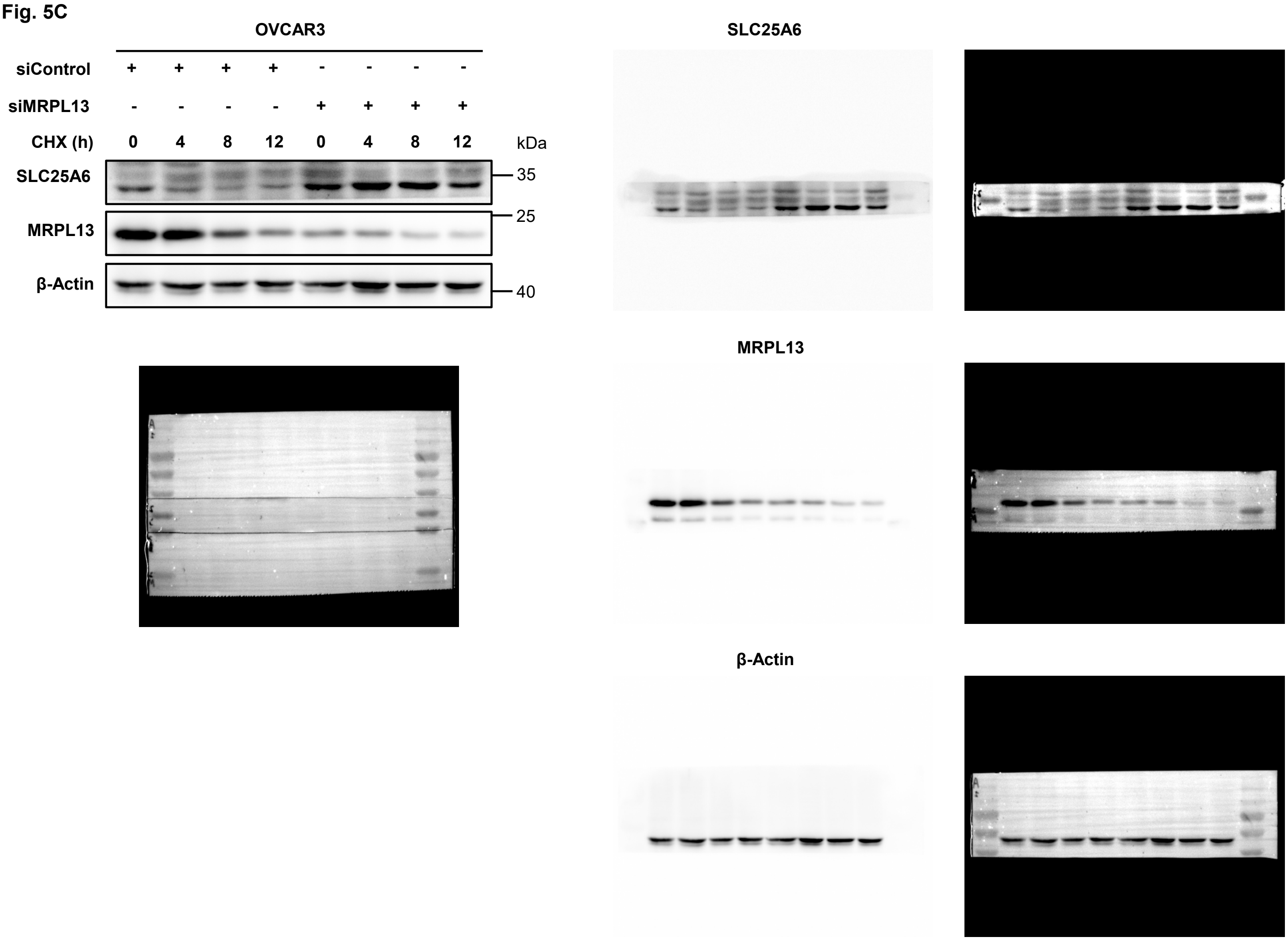
OVCAR3
siControl + + + + - - - -
siMRPL13 - - - - + + + +
CHX (h) 0 4 8 12 0 4 8 12
kDa
SLC25A6
35
MRPL13
25
β-Actin
40
SLC25A6
MRPL13
β-Actin

Fig. 5D

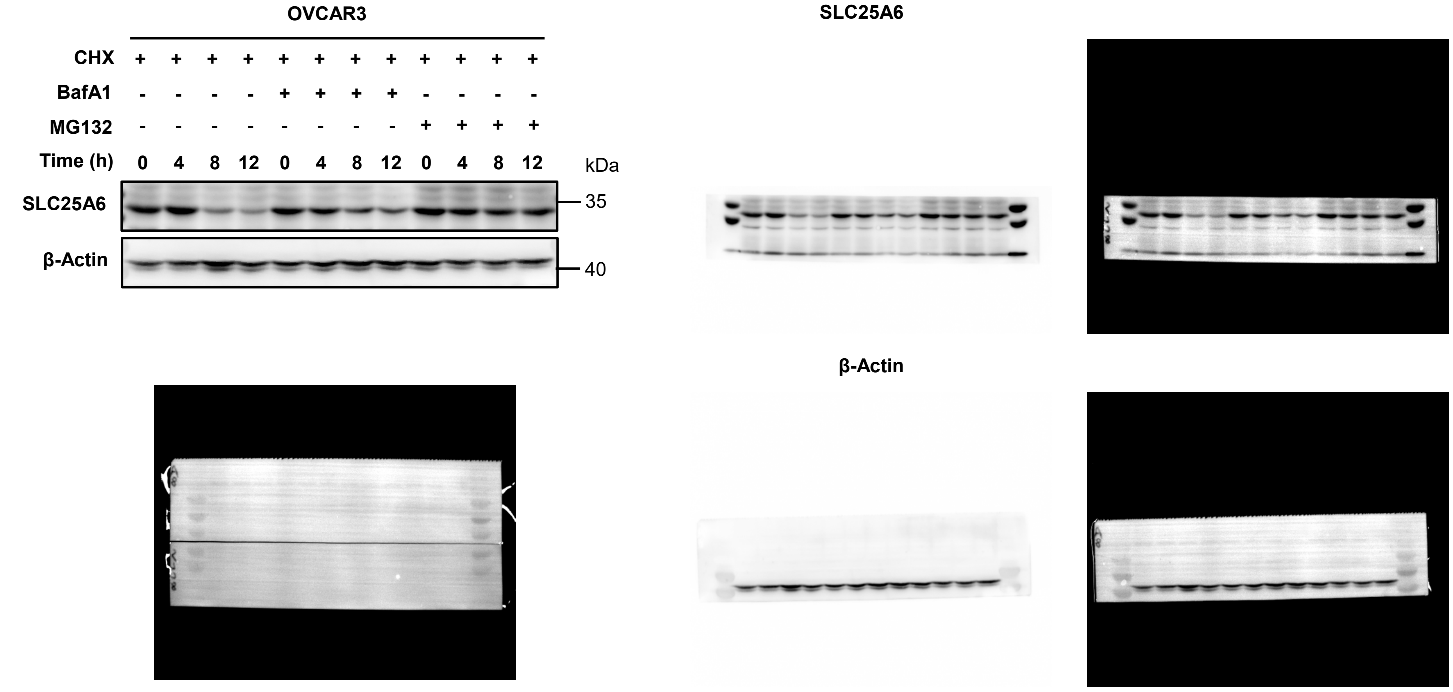
OVCAR3
CHX + + + + + + + + + + + +
BafA1 - - - - + + + + - - - -
MG132 - - - - - - - - + + + +
Time (h) 0 4 8 12 0 4 8 12 0 4 8 12
kDa
SLC25A6
35
β-Actin
40
SLC25A6
β-Actin

Fig. 5G

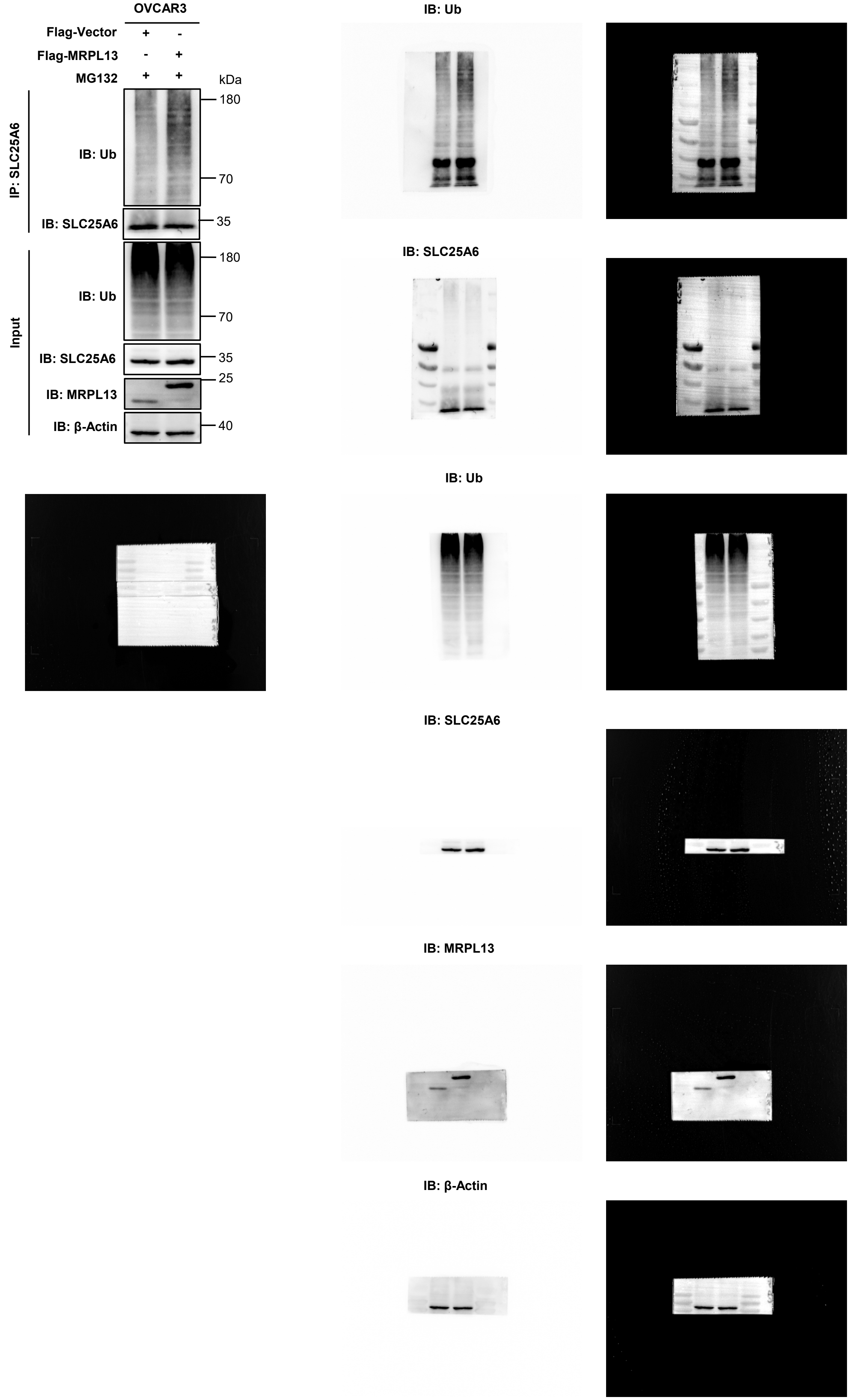
OVCAR3
Flag-Vector + -
Flag-MRPL13 - +
MG132 + +
kDa
IP: SLC25A6
IB: Ub
180
70
IB: SLC25A6
35
Input
IB: Ub
180
70
IB: SLC25A6
35
IB: MRPL13
25
IB: β-Actin
40
IB: Ub
IB: SLC25A6
IB: Ub
IB: SLC25A6
IB: MRPL13
IB: β-Actin

Fig. 5H

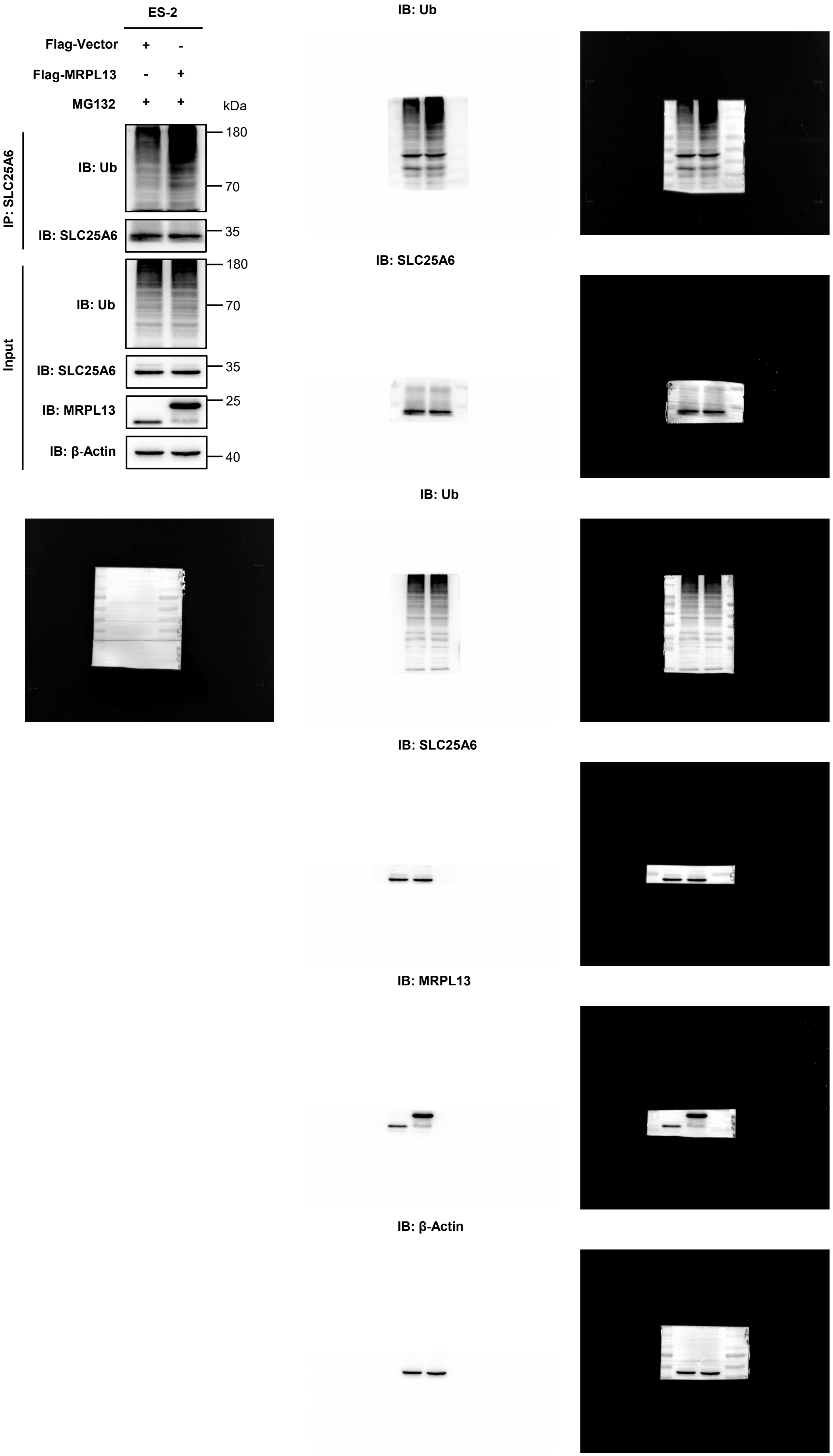
ES-2
Flag-Vector + -
Flag-MRPL13 - +
MG132 + +
kDa
IP: SLC25A6
IB: Ub
180
70
IB: SLC25A6
35
Input
IB: Ub
180
70
IB: SLC25A6
35
IB: MRPL13
25
IB: β-Actin
40
IB: Ub
IB: SLC25A6
IB: Ub
IB: SLC25A6
IB: MRPL13
IB: β-Actin

Fig. 5I

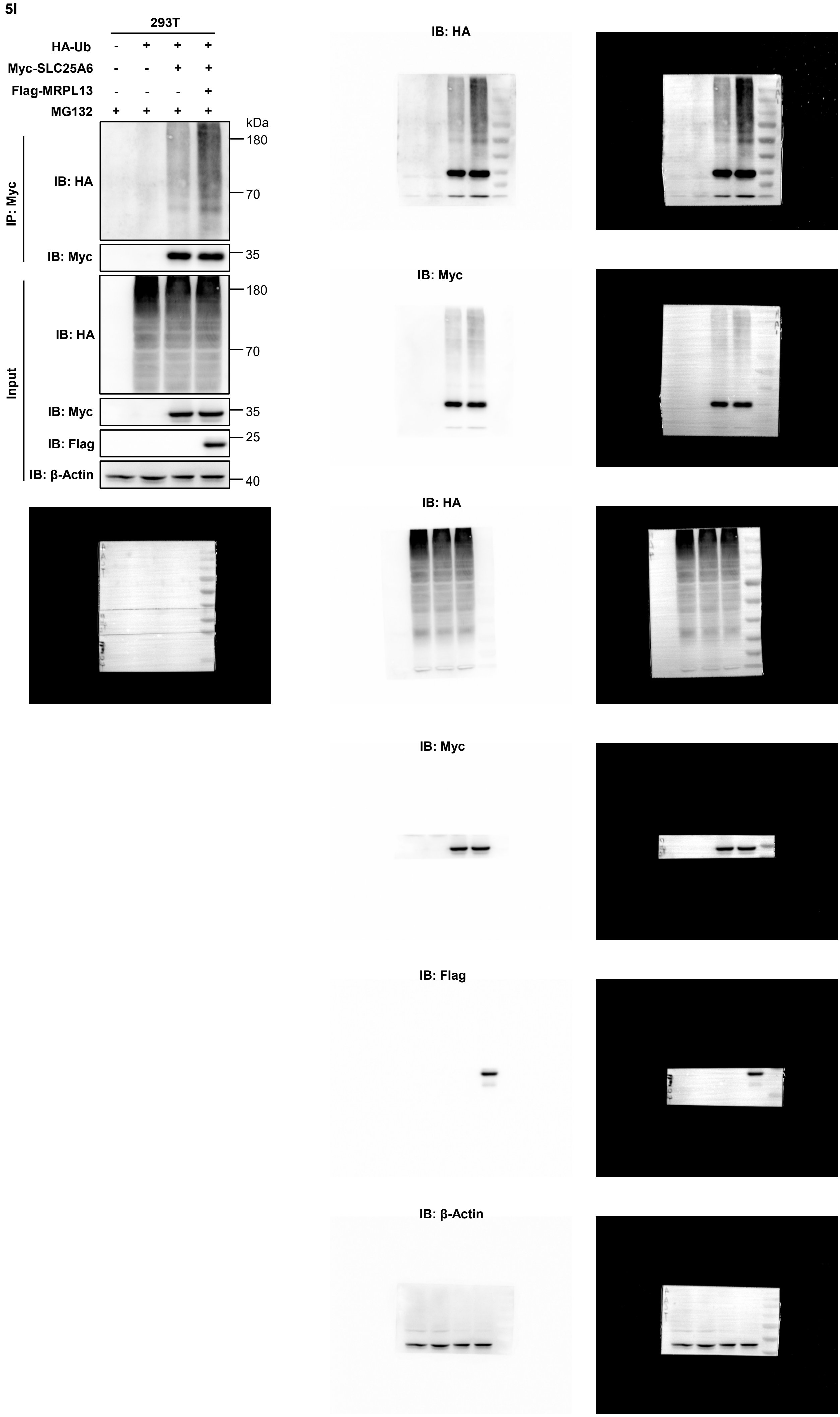
293T
HA-Ub
Myc-SLC25A6
Flag-MRPL13
MG132
kDa
180
70
35
180
70
35
25
40
IP: Myc
IB: HA
IB: Myc
Input
IB: HA
IB: Myc
IB: Flag
IB: β-Actin
IB: HA
IB: Myc
IB: HA
IB: Myc
IB: Flag
IB: β-Actin

Fig. 5J

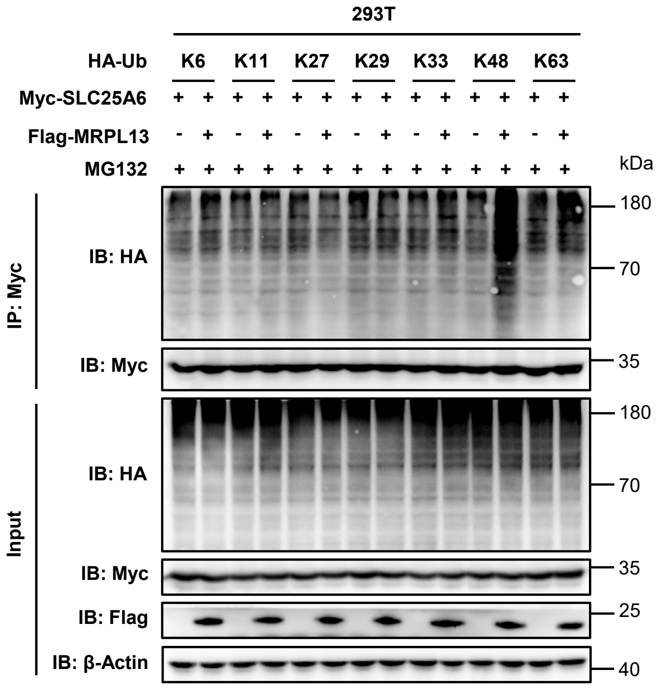

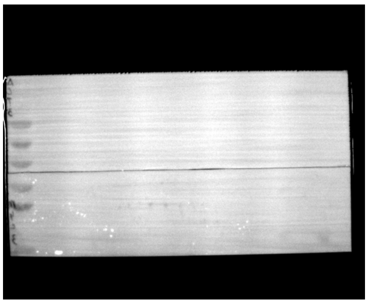

IB: HA

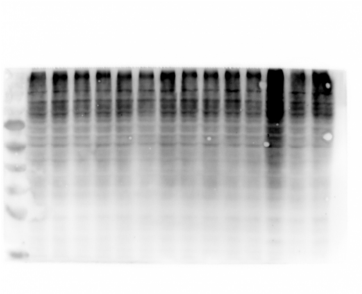

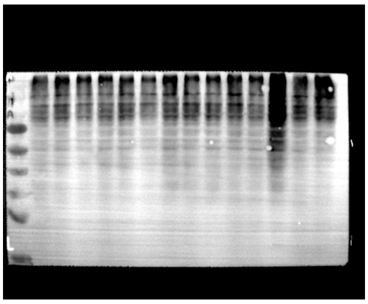

IB: Myc

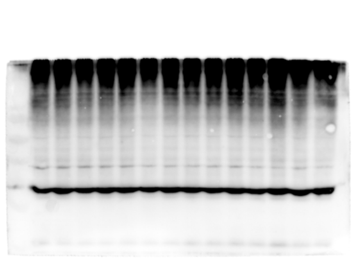

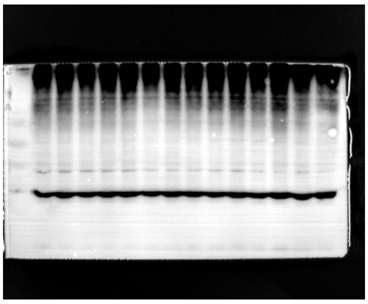

IB: HA

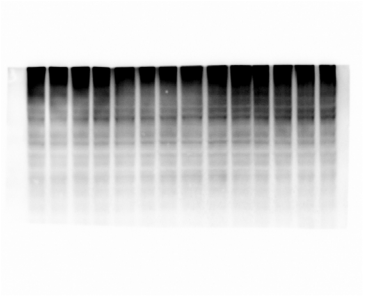

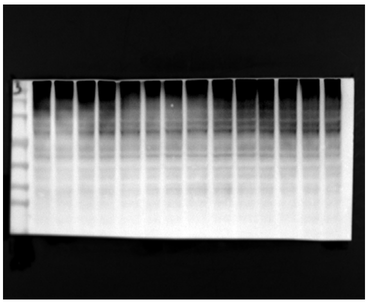

IB: Myc

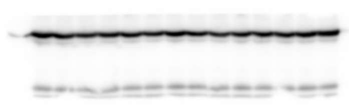

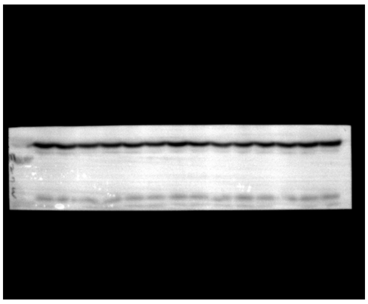

IB: Flag

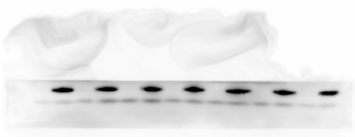

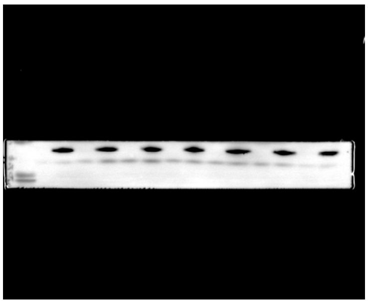

IB: β-Actin

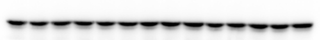

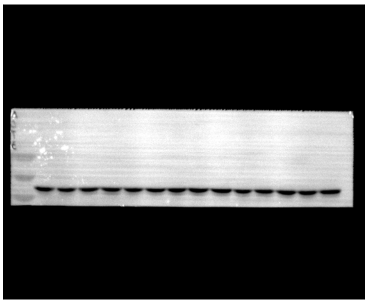

Fig. 6F

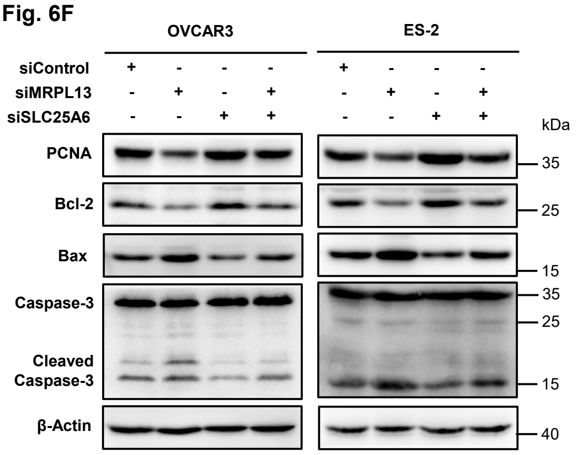

PCNA

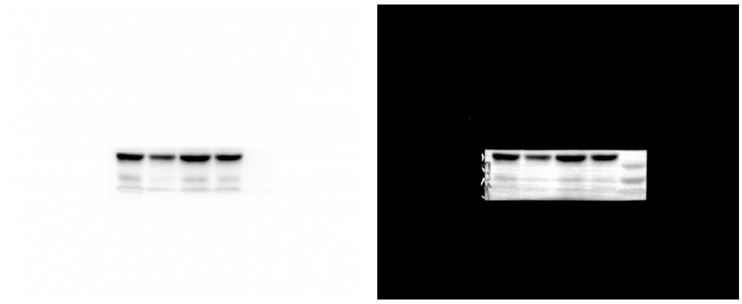

PCNA

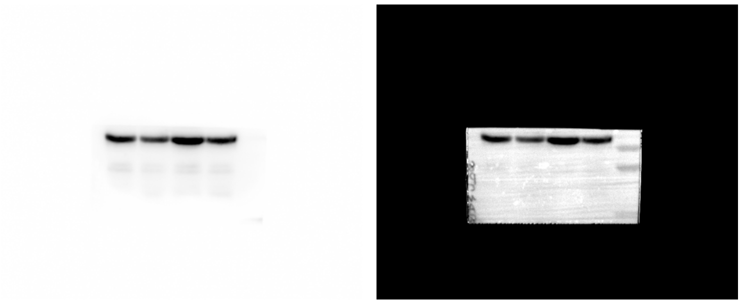

Bcl-2

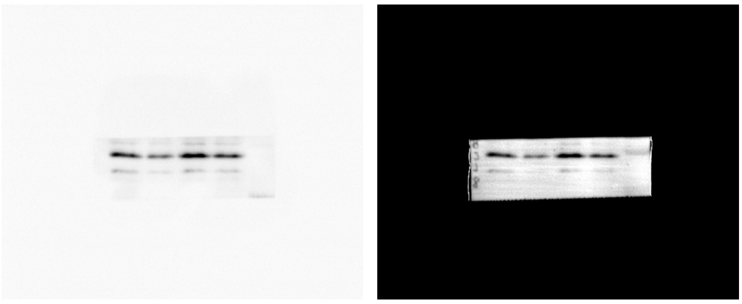

Bcl-2

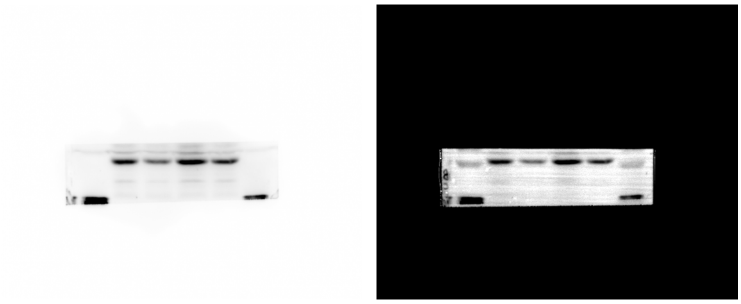

Bax

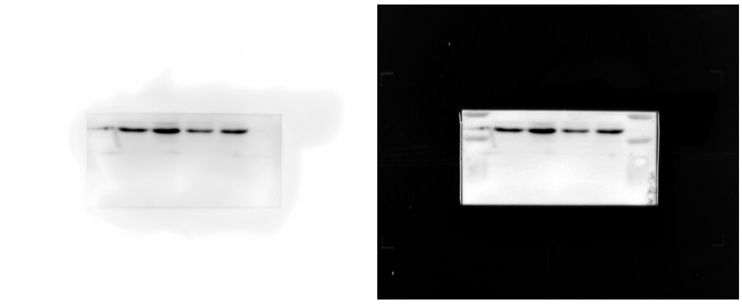

Bax

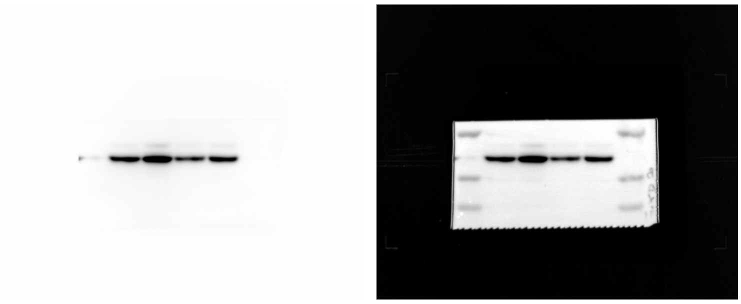

Caspase-3, Cleaved Caspase-3

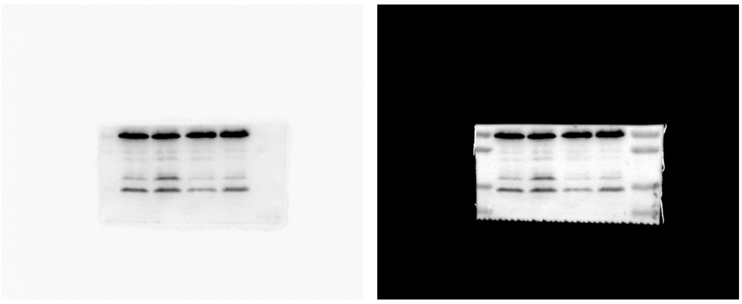

Caspase-3, Cleaved Caspase-3

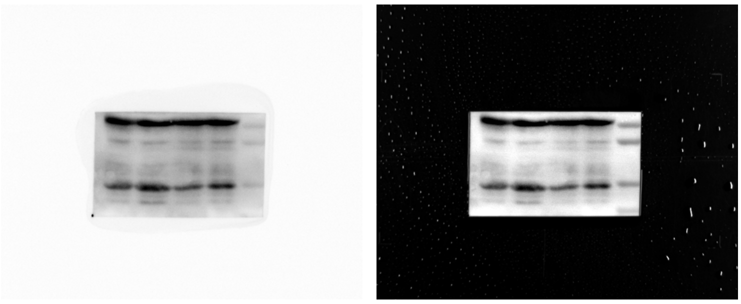

β-Actin

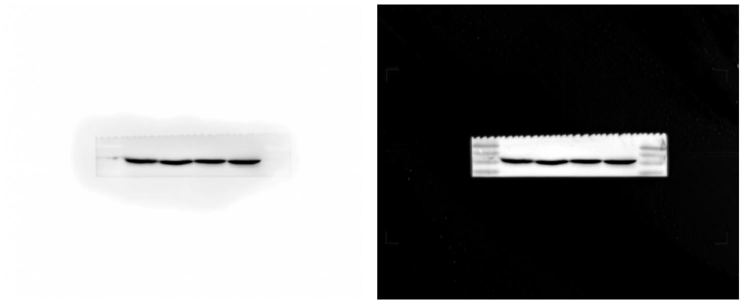

β-Actin

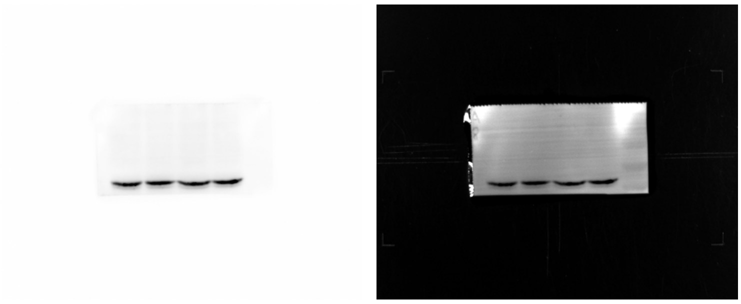

Fig. 7B

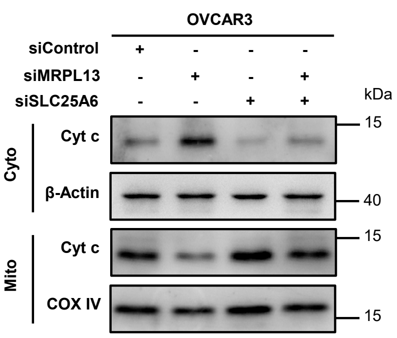

Cyt c

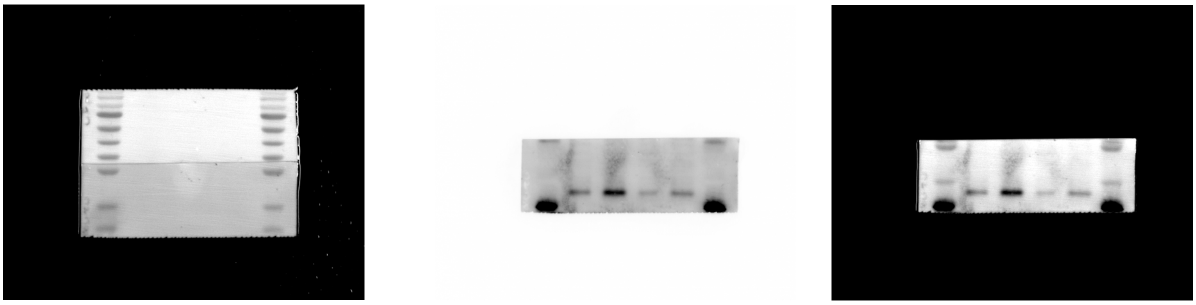

β-Actin

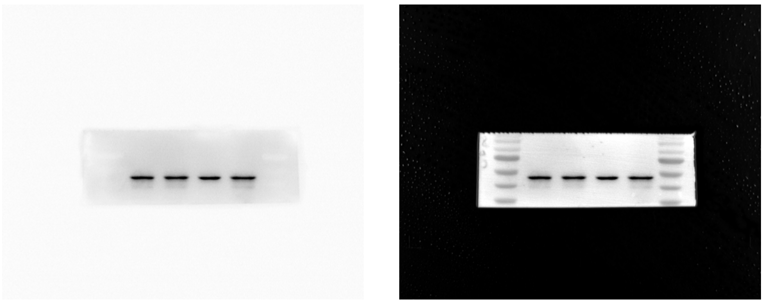

Cyt c

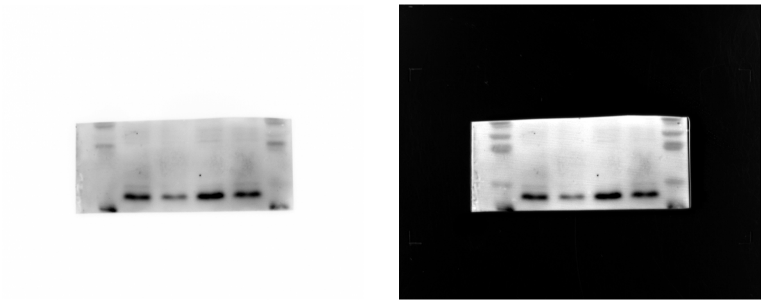

COX IV

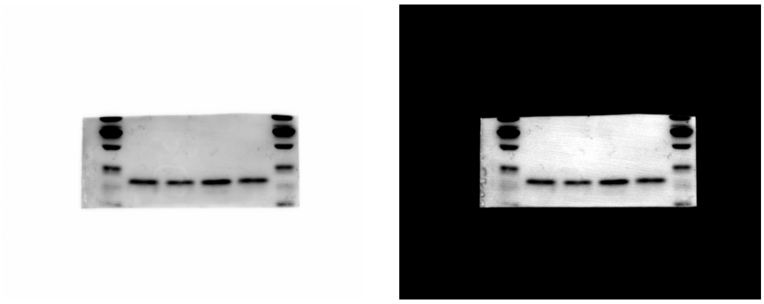

Supplementary Fig. 2A

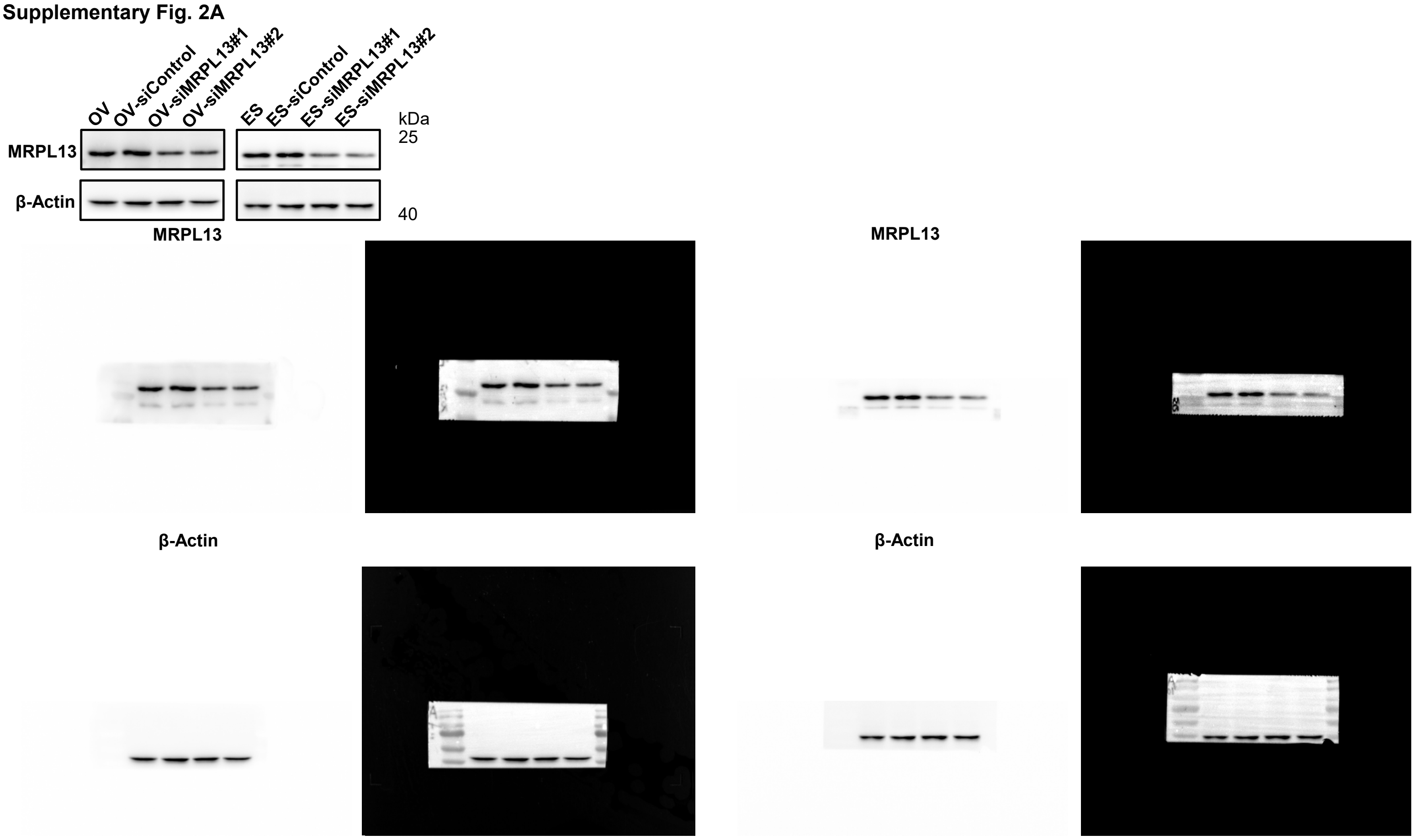

Supplementary Fig. 2B

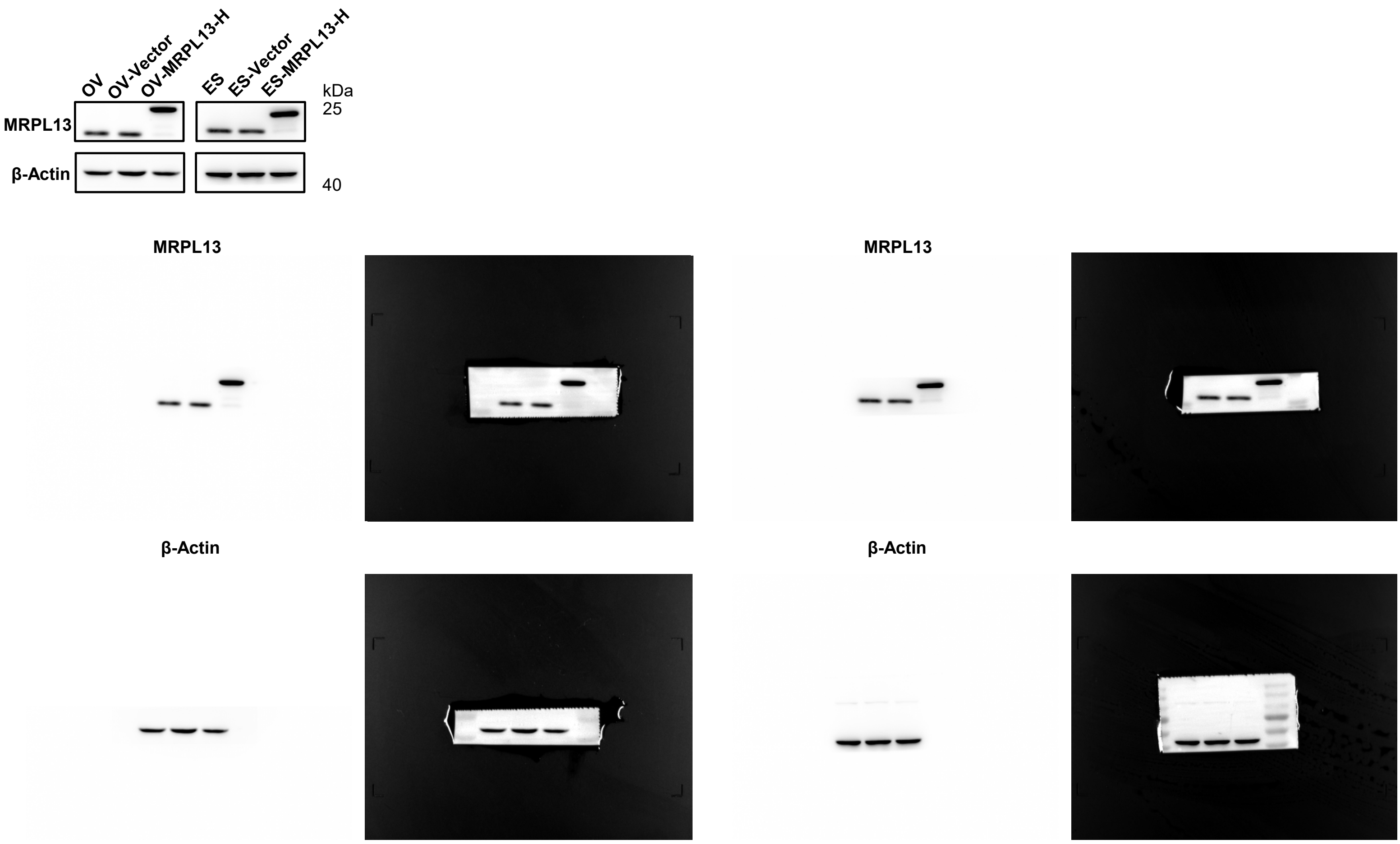

Supplementary Fig. 3G

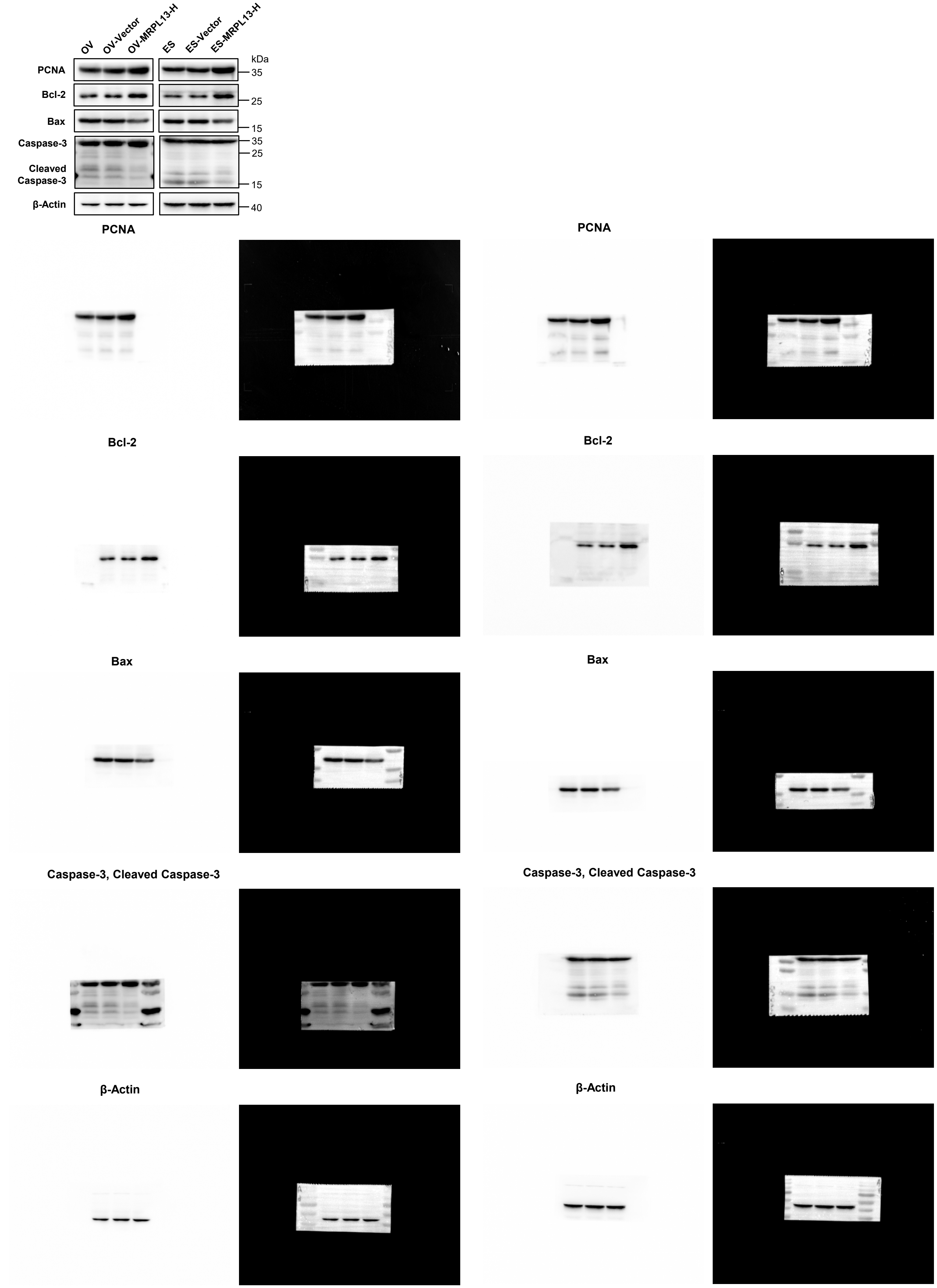

Supplement: Supplementary file 2 — Original Images for BlotsGels [file 41419_2025_7953_MOESM2_ESM.pdf]
